# Expanded sampling of New Zealand glass sponges (Porifera: Hexactinellida) provides new insights into biodiversity, chemodiversity, and phylogeny of the class

**DOI:** 10.7717/peerj.15017

**Published:** 2023-04-27

**Authors:** Martin Dohrmann, Henry M. Reiswig, Michelle Kelly, Sadie Mills, Simone Schätzle, Miriam Reverter, Natascha Niesse, Sven Rohde, Peter Schupp, Gert Wörheide

**Affiliations:** 1 Department of Earth and Environmental Sciences, Palaeontology & Geobiology, Ludwig-Maximilians-Universität München, Munich, Germany; 2 Biology Department, Natural History Section, University of Victoria, Victoria, British Columbia, Canada; 3 Coasts and Oceans National Centre, National Institute of Water & Atmospheric Research, Auckland, New Zealand; 4 Invertebrate Collection, National Institute of Water & Atmospheric Research, Wellington, New Zealand; 5 School of Biological and Marine Sciences, University of Plymouth, Plymouth, United Kingdom; 6 Institute for Chemistry and Biology of the Marine Environment, Carl von Ossietzky Universität Oldenburg, Wilhelmshaven, Germany; 7 Helmholtz Institute for Functional Marine Biodiversity, Carl von Ossietzky Universität Oldenburg, Oldenburg, Germany; 8 Bayerische Staatssammlung für Paläontologie und Geologie, Staatliche Naturwissenschaftliche Sammlungen Bayerns (SNSB), Munich, Germany; 9 GeoBio-CenterLMU, Ludwig-Maximilians-Universität München, Munich, Germany

**Keywords:** Biodiversity, Chemical fingerprinting, Glass sponges, Metabolome, Molecular phylogenetics, New Zealand

## Abstract

Glass sponges (Hexactinellida) constitute important parts of ecosystems on the deep-sea floor worldwide. However, they are still an understudied group in terms of their diversity and systematics. Here, we report on new specimens collected during RV *Sonne* expedition SO254 to the New Zealand region, which has recently emerged as a biodiversity hotspot for hexactinellids. Examination of the material revealed several species new to science or so far unknown from this area. While formal taxonomic descriptions of a fraction of these were published earlier, we here briefly report on the morphology of the remaining new species and use the collection to greatly expand the molecular phylogeny of the group as established with ribosomal DNA and cytochrome oxidase subunit I markers. In addition, we provide a chemical fingerprinting analysis on a subset of the specimens to investigate if the metabolome of glass sponges contains phylogenetic signal that could be used to supplement morphological and DNA-based approaches.

## Introduction

Glass sponges (Porifera: Hexactinellida) are globally important components of deep-sea benthic ecosystems, but the number of currently described extant species and genera (703 and 133, respectively; De Voogd et al., 2023) is unlikely to reflect the true diversity of the class (Reiswig, 2002a; Leys, Mackie & Reiswig, 2007). As exploration of the deep sea with modern equipment is steadily increasing in recent years, there has been a marked rise in new hexactinellid species, a trend that is likely to continue in the near future. The deep waters of the New Zealand (NZ) region (SW Pacific) provide a particularly striking example: While historical expeditions reported only few species from that region, it is now recognized as one of the biggest hotspots of glass sponge diversity, with 50 new species and 5 new genera described during the last ten years (Reiswig & Kelly, 2011; Reiswig & Kelly, 2017a; Reiswig & Kelly, 2017b; Reiswig & Kelly, 2018; Reiswig et al., 2021).

**Figure 1:**
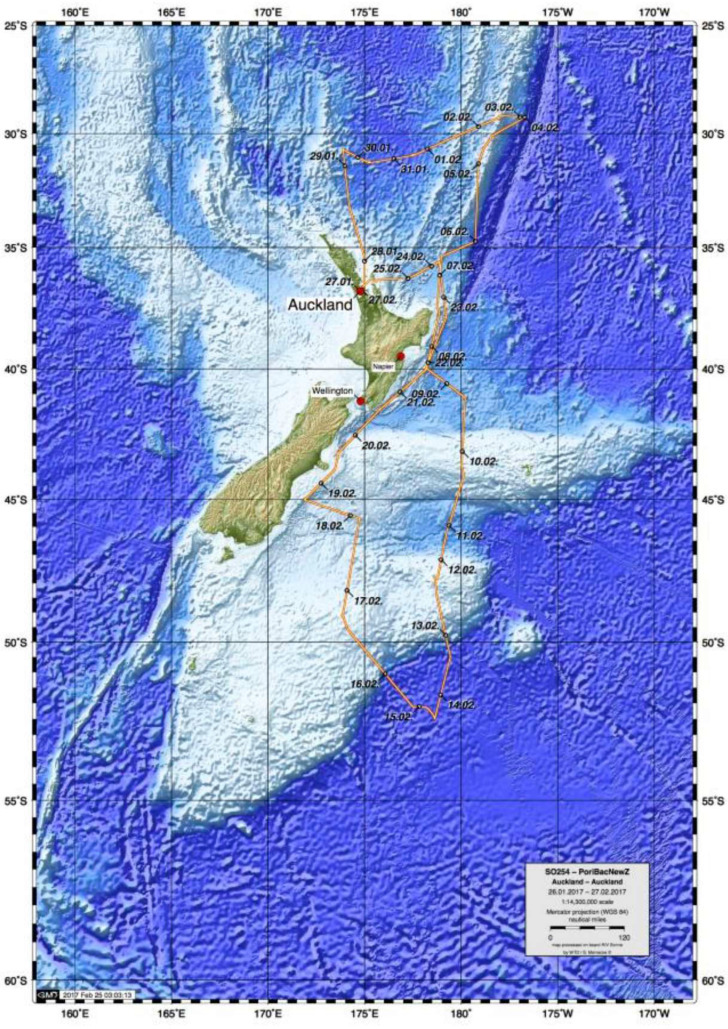
Map of study area showing trajectory of RV *Sonne* cruise SO254 with sampling stations.

In February 2017, the German RV *Sonne* expedition to New Zealand (cruise SO254), as part of Project *PoribacNewZ* of the University of Oldenburg and the LMU Munich, collected ∼100 new glass sponge specimens, using the Remotely Operated Vehicle (ROV) *Kiel 6000* of the GEOMAR Helmholtz Centre for Ocean Research Kiel (Fig. 1; Simon, 2017). Identification of these specimens through integration of molecular and morphological methods revealed the presence of 18 species, one subspecies, and two genera new to science, along with 18 previously described species that had not been included in molecular systematics studies thus far. Morphological descriptions of six of these species and one of the new genera were published recently, after the untimely passing of our co-author and eminent glass sponge specialist Henry M. Reiswig (Reiswig et al., 2021). Here, we report on the molecular phylogenetic results of the study, which increases the taxon sampling of Hexactinellida by 37 species and 12 genera (compared to Dohrmann, 2019) and present a preliminary morphological account of the new species, full taxonomic treatment of which will be published elsewhere. In addition to the molecular phylogeny, we also analyzed the metabolomic profiles of a subset of the investigated sponges to evaluate if chemical fingerprinting can corroborate the taxonomy of glass sponges.

## Materials & Methods

### Specimen collection and identification

Specimens, seafloor images, and videos were collected as part of Project *PoribacNewZ* of the Institute for Chemistry and Biology of the Marine Environment (ICBM), Carl von Ossietzky University of Oldenburg and the Department of Earth and Environmental Sciences, Palaeontology & Geobiology (DEES-PG) of the Ludwig-Maximilians-Universität (LMU) Munich, on the new German RV *Sonne* (voyage SO254) using the GEOMAR Helmholtz Centre for Ocean Research Kiel ROV *Kiel 6000* (Simon, 2017). Permit to collect marine organisms was provided by the Ministry of Foreign Affairs and Trade, Wellington, New Zealand.

In total, 101 glass sponge specimens were collected (Table S1). Subsamples were taken on board, stored in appropriate preservatives for morphological and molecular work, and shipped to DEES-PG at LMU Munich. Subsamples were also taken for chemical analysis by cutting 5 cm^3^ pieces from the specimens where possible, freezing them at −80 °C and shipping them to ICBM at the University of Oldenburg. Specimens were initially assigned to higher taxa by surveying underwater photographs. Subsequently, a molecular phylogenetic survey based on a mitochondrial 16S ribosomal DNA (16S) fragment (cf. Dohrmann et al., 2008) was performed at DEES-PG at the LMU Munich. This allowed assignment of all successfully sequenced specimens (*n* = 84) to families and, in some cases, to genera with reasonable confidence. Assignment to species was only successful for seven specimens, whose sequences were identical to those published for *Walteria leuckarti* Ijima, 1896 (Euplectellidae, three specimens) and *Aphrocallistes beatrix* Gray, 1858 (Aphrocallistidae, four specimens), respectively. Another specimen (NIWA126085) was tentatively identified as *Caulophacus (Caulophacus) arcticus* (Hansen, 1885) (Rossellidae) by this approach; however, this species’ arctic distribution casted some doubt on this identification, and morphological investigation revealed it as belonging to a new species (see below and Reiswig et al., 2021). The molecular survey further revealed the presence of multiple specimens for several other species (see Table S1 and full 16S phylogeny in Fig. S1).

For further integrative taxonomic and molecular phylogenetic study, 37 specimens (Table 1) were selected to maximize phylogenetic breadth and sequenced for additional mitochondrial (cytochrome oxidase subunit I [COI]) and nuclear (18S and 28S ribosomal DNA [18S, 28S]) markers previously established for the group (see Dohrmann et al., 2008; Dohrmann et al., 2012; Dohrmann et al., 2017 for methods). Preliminary morphological identifications of these specimens were done by MD by analyzing spicule content with light microscopy (LM) of bleach-digested tissue pieces. More thorough investigations and description of species new to science were then started to be performed by HMR using previously described methods (Reiswig & Kelly, 2011; Reiswig & Kelly, 2018). Descriptions of four of these species (as well as two re-descriptions of known species and two new species not selected for further sequencing here; all Rossellidae) were published previously, after the sad passing of Henry Reiswig in July 2020 (Reiswig et al., 2021). Formal descriptions of the remaining 14 species and one subspecies new to science will be provided elsewhere (work in progress).

**Table 1:** Glass sponge specimens from RV SONNE cruise SO254 selected for further molecular phylogenetic study.

Order	Family	Genus-Species	Authority	Main voucher	Other specimens^**^	16S	28S	18S	COI
Amphidiscosida	Hyalonematidae	*Hyalonema* n. sp.	description in prep.	126036	–	OX400575	OX400626 (3′)	OX400660	OX394279 (5′)
Amphidiscosida	Pheronematidae	*Pheronema* n. sp.	description in prep.	126138	126135	OX400572	OX400627 (3′)	–	–
Amphidiscosida	Pheronematidae	*Poliopogon* n. sp.	description in prep.	126337	–	OX400574	–	–	–
Sceptrulophora	Euretidae	*Eurete* n. sp. 1^*^	description in prep.	126028	–	OX400563	OX400624	OX400658	OX394277
Sceptrulophora	Euretidae	*Eurete* n. sp. 2^*^	description in prep.	126276	–	OX400564	OX400625	OX400659	OX394278 (3′)
Sceptrulophora	Farreidae	*Farrea ananchorata*	Reiswig & Kelly (2011)	126320	126278	OX400570	OX400622	OX400656	–
Sceptrulophora	Farreidae	*Farrea occa* n. ssp.	Bowerbank, 1862; subspecies description in prep.	126307	–	OX400569	OX400621	OX400657	–
Sceptrulophora	Farreidae	*Farrea* n. sp.	description in prep.	126004	–	OX400568	OX400620 (5′)	OX400653	OX394276 (5′)
Sceptrulophora	Farreidae	*Farrea raoulensis*	Reiswig & Kelly (2011)	126015	–	OX400567	–	OX400654	–
Sceptrulophora	Farreidae	*Farrea similaris*	Reiswig & Kelly (2011)	126345	126011	OX400565	OX400623	OX400655	OX394275 (5′)^***^
Lyssacinosida	Euplectellidae	*Amphidiscella abyssalis* ^*^	Reiswig & Kelly (2018)	126030	126031, 126032, 126033	OX400549	OX400617	OX400645	OX394272
Lyssacinosida	Euplectellidae	*Amphidiscella sonnae* ^*^	Reiswig & Kelly (2018)	126034	–	OX400553	OX400616	OX400646	OX394271 (5′)
Lyssacinosida	Euplectellidae	*Amphoreus schuppi* ^*^	Reiswig & Kelly (2018)	126035	–	OX400554	OX400613	OX400650	OX394274 (5′)
Lyssacinosida	Euplectellidae	*Bolosoma cyanae*	Tabachnick & Lévi, 2004	126339	126340, 126341, 126346	OX400523	OX400618	OX400652	OX394270^***^
Lyssacinosida	Euplectellidae	*Bolosoma meridionale*	Tabachnick & Lévi, 2004	126121	–	OX400555	OX400619	OX400651	–
Lyssacinosida	Euplectellidae	*Saccocalyx tetractinus*	Reiswig & Kelly (2018)	126323	126012, 126029, 126321, 126322	OX400546	OX400612	OQ301566	OX394269^***^
Lyssacinosida	Euplectellidae	Trychella kermadecensis^*^	Reiswig & Kelly (2018)	126125	–	OX400558	OX400614 (5′)	OX400648 (5′)	–
Lyssacinosida	Euplectellidae	*Trychella* n. sp.^*^	description in prep.	126306	–	OX400557	OX400615	OX400649 (5′)	OX394273
Lyssacinosida	Euplectellidae	*Corbitella* n. sp.	description in prep.	126122	126123, 126350	OX400539	OX400609	OX400643 (5′)	OX394268^***^
Lyssacinosida	Euplectellidae	*Corbitella plagiariorum*	Reiswig & Kelly (2018)	126162	126163, 126164, 126165, 126168	OX400535	OX400610	OX400644	OX394267
Lyssacinosida	Euplectellidae	Dictyaulus hydrangeaformis^*^	Reiswig & Kelly (2018)	126124	–	OX400556	OX400611	OX400647 (5′)	–
Lyssacinosida	Euplectellidae	*Regadrella pedunculata*	Reiswig & Kelly (2018)	126299	–	OX400527	OX400607	OX400641 (5′)	OX394265
Lyssacinosida	Euplectellidae	*Regadrella okinoseana*	Ijima, 1896	126166	126296, 126297, 126298	OX400531	OX400608	OX400642 (5′)	OX394266
Lyssacinosida	Aulocalycidae	*Aulocalyx* n. sp.^*^	description in prep.	126318	126176, 126301	OX400520	OX400596	OX400631	OX394257 (5′)
Lyssacinosida	Aulocalycidae	Aulocalyx serialis^*^	Dendy, 1916	126349	126017	OX400518	OX400597^***^	OX400632	OX394258 (5′)
Lyssacinosida	Aulocalycidae	*Rhabdodictyum* n. sp.^*^	description in prep.	126083	–	OX400522	OX400598	OX400633	–
Lyssacinosida	Leucopsacidae	*Chaunoplectella* n. sp.^*^	description in prep.	126325	–	OX400515	OX400593 (5′)	OX400628	OX394256
Lyssacinosida	Leucopsacidae	*Leucopsacus distantus* ^*^	Tabachnick & Lévi, 2004	126158	126300, 126317	OX400512	OX400595	OX400630	OX394254 (5′)^***^
Lyssacinosida	Leucopsacidae	*Leucopsacus* n. sp.^*^	description in prep.	126063	–	OX400516	OX400594	OX400629	OX394255 (5′)
Lyssacinosida	Rossellidae	Lanuginellinae n. gen. n. sp.^*^^,^^a^	description in prep.	126169	126302, 126310, 126315, 126319	OX400508	OX400599 (5′)^***^	OX400634^***^	OX394263 (5′)^***^
Lyssacinosida	Rossellidae	*Lophocalyx* n. sp.	description in prep.	126005	126014	OX400511	OX400602 (3′)	OX400635 (5′)	OX394262 (5′)^***^
Lyssacinosida	Rossellidae	*Caulophacus (Caulophacus) serpens*	Reiswig et al. (2021)	126084	–	OX400509	OX400600 (5′)	OX400636	–
Lyssacinosida	Rossellidae	*Caulophacus (Caulophacus) discohexaster*	Tabachnick & Lévi, 2004	126342	126343	OX400506	OX400601	OX400637	OX394264 (3′)
Lyssacinosida	Rossellidae	*Bathydorus poculum*	Reiswig et al. (2021)	126338	–	OX400504	OX400606	OX400640	–
Lyssacinosida	Rossellidae	*Nubes poculiformis* ^*^	Reiswig et al. (2021)	126016	–	OX400496	OX400604	OX400638	OX394259
Lyssacinosida	Rossellidae	*Nubes tubulata* ^*^	Reiswig et al. (2021)	126159	126160	OX400497	OX400605	OX400639	OX394260 (5′)
Lyssacinosida	Rossellidae	*Scyphidium australiense* ^*^	Tabachnick, Janussen & Menschenina, 2008	126237	–	OX400493	OX400603 (3′)^***^	–	OX394261 (5′)^***^

**Notes.**

Associated European Nucleotide Archive (ENA) accession numbers given for each marker; if only one half could be sequenced this is indicated by “(5’)” or “(3’)”.

* Genus sequenced for the first time.

** Identical 16S sequences indicate that these specimens are from the same species (see Fig. S1).

*** Sequences obtained from other specimen(s).

a During the production stage of this article, we became aware of the publication by Tabachnick, Menshenina & Ehrlich (2023), who abolished the subfamilies of Rossellidae. While we principally support this move, we wish to point out that Lanuginellinae remains valid as a phylogenetic group (clade), given its strong molecular support (see also Dohrmann et al., 2017: p. 27 for a morphological diagnosis).

### Molecular phylogenetics

Newly generated sequences (Table 1) and sequences of *Rhizophyta yapensis* (Shen et al., 2019) were manually added to the alignments provided by Dohrmann (2019) using AliView (Larsson, 2014); duplicates and some problematic sequences (cf. Dohrmann, 2019) were removed. In case of duplicates, *i.e.,* multiple specimens of the same species (mostly from Vargas et al., 2017; Kersken et al., 2018a), the specimen with the least amount of missing data was chosen as that species’ representative. Phylogenetic analyses were performed in maximum likelihood (ML) and Bayesian inference (BI) frameworks (Ronquist et al., 2012; Stamatakis, 2014) using partitioned DNA/RNA substitution models as described in Dohrmann et al. (2017). For BI, chains were run for 10 ×10^6^ generations and the first 10% of samples discarded as burnin. Alignments and trees are deposited in a GitHub repository (https://github.com/PalMuc/SONNE_Hexactinellida).

### Chemical fingerprinting

For the chemical extraction, 21 Hexactinellida specimens belonging to eight families with 12 different genera were analyzed, representing a subsample of the specimens included in the molecular phylogenetic study. Specimens were used for chemical fingerprinting if vouchers stored at −80 °C were available, which was not always the case due to limited sponge material. Samples were freeze-dried and ground to a homogeneous powder. For chemical extractions, 1 g of each sample was extracted in 10 mL high performance liquid chromatography (HPLC)-grade MeOH/EtoAc (1:1) on a shaking plate for 30 min and then centrifuged. The supernatant was pipetted, transferred to a second vial, and evaporated to dryness under vacuum. The extraction was repeated two more times, each time with 10 mL MeOH/EtoAc (1:1). Subsequently, the combined extracts were dissolved in HPLC-grade MeOH, filtered (filter size 0.2 µm PTFE membrane filter, VWR international, USA) and brought to a final concentration of 1 mg/L.

For UPLC/MS analysis, an ACQUITY Ultra Performance Liquid Chromatography (UPLC) H-Class system (Waters Co., Milford, MA, USA) coupled with a Synapt G2-Si HDMS high-resolution Q-ToF-MS (Waters Co., Manchester, United Kingdom) was used to obtain the mass spectra. Chromatographic separation was performed using a Waters Acquity BEH C18 column (1.7 µm, 2.1 mm × 50 mm). Analytes were eluted at a flow rate of 0.6 mL min^−1^ using a linear elution gradient of H_2_O (100%, eluent A) to acetonitrile (ACN, 100%, eluent B) both with 0.1% formic acid. The initial condition was 100% A held for 0.5 min, followed by a linear gradient to 100% B in 19.5 min. The column was then washed with 100% B for 9.5 min and subsequently returned and held for 2.9 min to the initial conditions (100% eluent A) to equilibrate the column for the following run. The column temperature was set at 40 °C and the injection volume was 1 µL.

The mass spectrometer was calibrated and handled as described in Kamyab et al. (2020: p. 11). Samples were injected using a Latin Square design to reduce systematic technical errors. At the beginning of the sequence three blank (*i.e.,* methanol) samples were injected, followed by five quality control (QC) samples (*i.e.,* pool of biological samples). QC samples were then equidistantly (every five injections) analyzed throughout the sequence. At the end of the sequence, two QC samples and two blank samples were analyzed.

The open-source tool msConvert from the open-source ProteoWizard library (Chambers et al., 2012) was used to convert the raw data into mzML files. mzML files were processed using the XCMS online tool (version 2.7.2., XCMS version 1.47.3.) (Forsberg et al., 2018) to detect, deconvolute and align features, which provided a matrix containing the retention time, m/z value and integrated peak areas of the identified features. The CentWave algorithm was used for peak detection (maximum m/z deviation = 15 ppm, peak width = c(2,30), mzdiff = 0.01), and obiwarp was used for peak alignment and retention time correction (profStep = 0.5, bw = 5, mzwid = 0.05). Features that had (1) a coefficient of variation higher than 20% among the QC samples, (2) blank intensities that were over 50% of those observed in biological samples (at least one sample), and (3) peak area intensities <10,000 in all sample groups were removed. This resulted in a dataset that contained 4,742 features. A dataset only containing major features (*i.e.,* >5,000,000 integrated peak area in at least one of the sample groups, 112 features) was also generated.

All numerical analyses were performed in R version 4.1.2. (R Core Team, 2021). Integrated peak areas were log transformed prior to multivariate analyses. Non-metric Multidimensional Scaling analysis based on Euclidean distance (NMDS, function metaMDS from the vegan package, without auto-transformation), was used to visualize the similarity in the metabolic fingerprints of the different sponge species. An Analysis of Similarities (ANOSIM) using Euclidean distance was used to test for statistical difference in the metabolic profiles of the different sponge groups (*i.e.,* taxonomic families). A hierarchical cluster analysis (function hclust, Euclidean distance) was used to classify sponge species based on the similarity of their metabolomes. The “Average” algorithm was chosen after analysis of the cophenetic correlation coefficient, and the Kelley-Gardner-Sutcliffe (KGS) penalty function (maptree R package) was used to prune the dendrogram and obtain the optimal number of clusters.

## Results & Discussion

### Systematic account

Following the World Porifera Database (De Voogd et al., 2023).

**Table:** 

PORIFERA Grant, 1836
HEXACTINELLIDA Schmidt, 1870
AMPHIDISCOPHORA Schulze, 1886
AMPHIDISCOSIDA Schrammen, 1924
HYALONEMATIDAE Gray, 1857
***Hyalonema*** Gray, 1832
*Hyalonema* **n.** **sp.**
PHERONEMATIDAE Gray, 1870
***Pheronema*** Leidy, 1868
*Pheronema* **n.** **sp.**
***Poliopogon*** Thomson, 1877
*Poliopogon* **n. sp.**
HEXASTEROPHORA Schulze, 1886
SCEPTRULOPHORA Mehl, 1992
APHROCALLISTIDAE Gray, 1858
***Aphrocallistes*** Gray, 1858
*Aphrocallistes beatrix beatrix* Gray, 1858*
EURETIDAE Zittel, 1877
***Eurete*** Semper, 1868**
*Eurete* **n. sp. 1**
*Eurete* **n. sp. 2**
FARREIDAE Gray, 1872
***Farrea*** Bowerbank, 1862
*Farrea ananchorata* Reiswig & Kelly, 2011
*Farrea occa* Bowerbank, 1862 **n. ssp.**
*Farrea raoulensis* Reiswig & Kelly, 2011
*Farrea similaris* Reiswig & Kelly, 2011
*Farrea* **n. sp.**

**Table:** 

LYSSACINOSIDA Zittel, 1877
EUPLECTELLIDAE Gray, 1867
BOLOSOMINAE Tabachnick, 2002
***Amphidiscella*** Tabachnick & Lévi, 1997
*Amphidiscella abyssalis* Reiswig & Kelly, 2018
*Amphidiscella sonnae* Reiswig & Kelly, 2018
***Amphoreus*** Reiswig & Kelly, 2018
*Amphoreus schuppi* Reiswig & Kelly, 2018
***Bolosoma*** Ijima, 1904
*Bolosoma cyanae* Tabachnick & Lévi, 2004
*Bolosoma meridionale* Tabachnick & Lévi, 2004
***Saccocalyx*** Schulze, 1896
*Saccocalyx tetractinus* Reiswig & Kelly, 2018
***Trychella*** Reiswig & Kelly, 2018
*Trychella kermadecensis* Reiswig & Kelly, 2018
*Trychella* **n. sp.**
CORBITELLINAE Gray, 1872
***Corbitella*** Gray, 1867
*Corbitella plagiariorum* Reiswig & Kelly, 2018
*Corbitella* **n. sp.**
***Dictyaulus*** Schulze, 1896
*Dictyaulus hydrangeaformis* Reiswig & Kelly, 2018
***Regadrella*** Schmidt, 1880
*Regadrella okinoseana* Ijima, 1896
*Regadrella pedunculata* Reiswig & Kelly, 2018
***Walteria*** Schulze, 1886
*Walteria leuckarti* Ijima, 1896*
AULOCALYCIDAE Ijima, 1927
***Aulocalyx*** Schulze, 1886
*Aulocalyx serialis* Dendy, 1916**
*Aulocalyx* **n. sp.**
***Rhabdodictyum*** Schmidt, 1880**
*Rhabdodictyum* **n. sp.**
LEUCOPSACIDAE Ijima, 1903
***Chaunoplectella*** Ijima, 1896**
*Chaunoplectella* **n. sp.**
***Leucopsacus*** Ijima, 1898
*Leucopsacus distantus* Tabachnick & Lévi, 2004
*Leucopsacus* **n. sp.**
ROSSELLIDAE Schulze, 1885

**Table:** 

***Caulophacus (Caulophacus)*** Schulze, 1886
*Caulophacus (Caulophacus) discohexaster* Tabachnick & Lévi, 2004
*Caulophacus (Caulophacus) serpens* Reiswig, Dohrmann & Kelly, 2021
*Caulophacus (Caulophacus) ramosus* Reiswig, Dohrmann & Kelly, 2021*
***Lophocalyx*** Schulze, 1887
*Lophocalyx* **n. sp.**
Lanuginellinae **n. gen.**
Lanuginellinae **n. gen. n. sp.**
***Bathydorus*** Schulze, 1886
*Bathydorus poculum* Reiswig, Dohrmann & Kelly, 2021
***Nubes*** Reiswig, Dohrmann & Kelly, 2021
*Nubes poculiformis* Reiswig, Dohrmann & Kelly, 2021
*Nubes tubulata* Reiswig, Dohrmann & Kelly, 2021
***Scyphidium*** Schulze, 1900
*Scyphidium australiense* Tabachnick, Janussen & Menschenina, 2008
*Scyphidium variospinosum* Reiswig, Dohrmann & Kelly, 2021*
*Only sequenced for 16S
**New record for NZ waters

### Taxonomic notes

**Figure 2:**
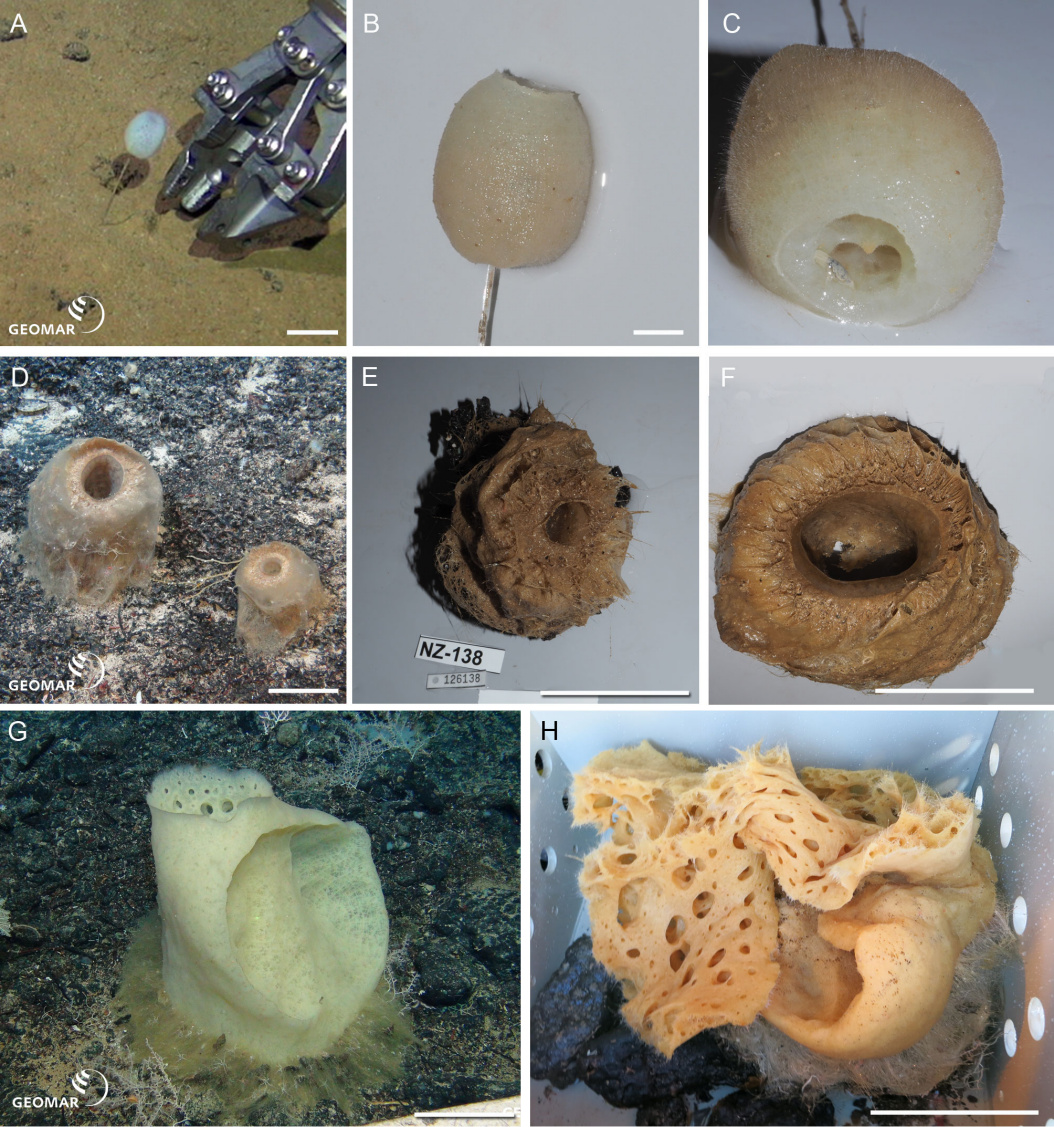
New species of Amphidiscosida. *Hyalonema* n. sp. (NIWA 126036) from the abyssal basin between Three Kings and Colville Ridges, northern New Zealand, 4,158 m: (A) Sponge *in situ* next to ROV collection arm, scale = 40 mm; (B) Deck image showing the globular, symmetrical body on a fine spicule-rope stem, scale = 10 mm; (C) Deck image showing sponge apex with compound osculum. *Pheronema* n. sp. from the Southern Kermadec Ridge, northeastern North Island, New Zealand, 1,169 m: (D) *In situ* image of NIWA 126135 (left) and NIWA 126138 (right), scale = 100 mm; (E) Deck image of NIWA 126138, scale = 100 mm; (F) Deck image of NIWA 126135, scale = 100 mm. *Poliopogon* n. sp. (NIWA 126337) from the Southern Kermadec Ridge, northeastern North Island, New Zealand, 1,150 m: (G) Sponge *in situ* showing inhalant and exhalant cones and basal attachment fringe, scale = 100 mm; (H) Deck image, scale = 100 mm. Images (A, D, and G) captured by ROV Team GEOMAR, ROV *Kiel 6000* onboard RV *Sonne* (voyage SO254), courtesy of Project PoribacNewZ, GEOMAR, and ICBM.

**Figure 3:**
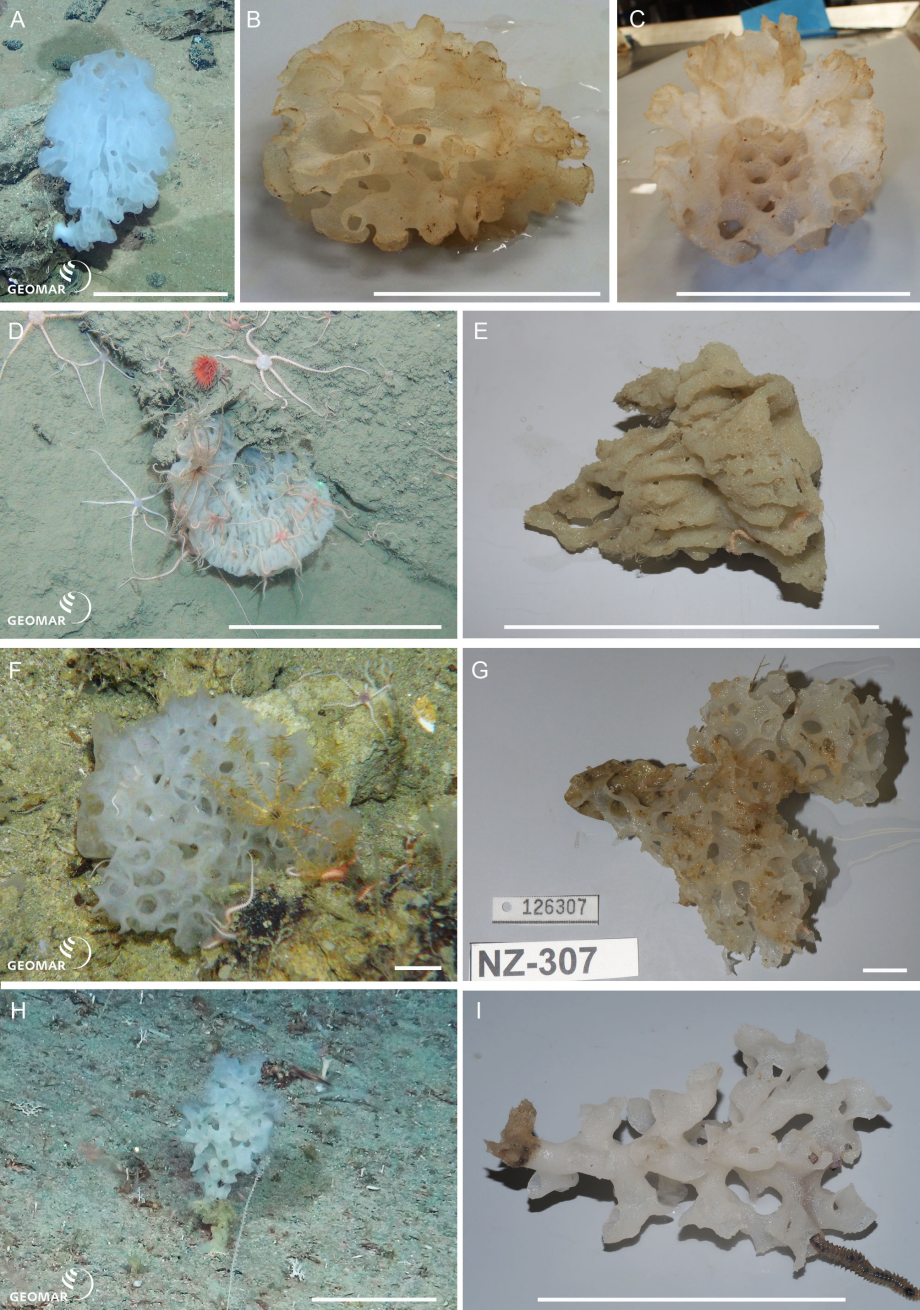
New species of Sceptrulophora. *Eurete* n. sp. 1 (NIWA 126028) from the abyssal basin between Three Kings and Colville Ridges, northern New Zealand, 4,160 m: (A) *In situ* image showing the pedunculate, cavernous form, scale = 10 cm; (B) Deck image showing the lacy, cavernous body; (C) Deck image showing the wide perforated atrium, scale = 10 cm. *Eurete* n. sp. 2 (NIWA 126276) from Wairarapa Slope, southeastern North Island, New Zealand, 1,663 m: (D) *In situ* image of the shallow-chalice shaped sponge, scale = 10 cm; (E) Deck image showing the sculpted, ridged undersides, scale = 10 cm. *Farrea occa* n. ssp. (NIWA 126307) from Seamount 986, off Hawke Bay, eastern North Island, New Zealand, 893 m: (F) *In situ* image showing the thick, fan-shaped, honeycombed body, scale = 1 cm; (G) Deck image, scale = 1 cm. *Farrea* n. sp. (NIWA 126004) from Seamount 986, off Hawke Bay, eastern North Island, New Zealand, 893 m: (H) *In situ*, scale = 10 cm; (I) Deck image showing hollow, tubular branches with flared termini, scale = 10 cm. Image A reproduced from Reiswig & Kelly (2018): 148, seafloor image 2, with permission from the *NIWA Biodiversity Memoir* Series editors. Images A, D, F, H captured by ROV Team GEOMAR, ROV *Kiel 6000* onboard RV *Sonne* (voyage SO254), courtesy of Project PoribacNewZ, GEOMAR, and ICBM.

**Figure 4:**
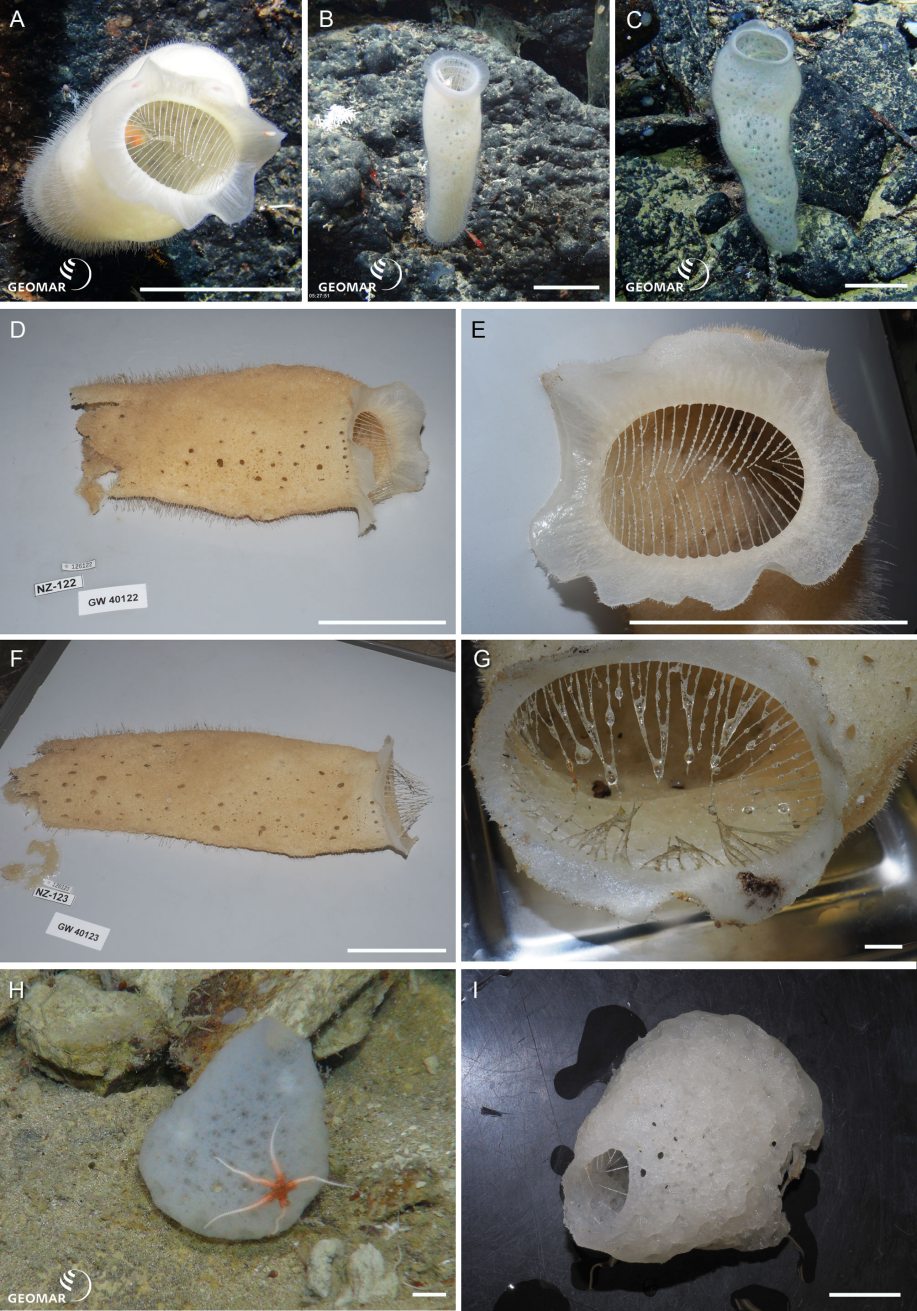
New species of Euplectellidae. *Corbitella* n. sp. from the Southern Kermadec Ridge, northeastern North Island, New Zealand, 1,164–1,205 m: (A) *In situ* image of NIWA 126122, showing the hispid body wall, and the delicate corona of marginal spicules projecting in a broad, thin, wavy cuff on the upper body wall, with eyelash-like hexactine parietal prostalia projecting from the rim. Note the resident Venus shrimp (*Spongicoloides* sp.), scale = 100 mm; (B) *In situ* image of NIWA 126123, with narrow upper body wall cuff, scale = 100 mm; (C) *In situ* image of NIWA 126350, with bulbous body and narrow upper body wall cuff, scale = 100 mm; (D) Deck image of NIWA 126122, scale = 100 mm; (E) Closeup of marginal cuff and eyelash-like hexactine parietal prostalia projecting from the rim, scale = 100 mm; (F) Deck image of NIWA 126123, scale = 100 mm; (G) Upper body wall and margin of NIWA 126350, scale = 10 mm. *Trychella* n. sp. (NIWA 126306) from Seamount 986, off Hawke Bay, eastern North Island, New Zealand, 893 m: (H) *In situ* image, scale = 10 mm; (I) Deck image showing tooth-like marginal spicules projecting across the apex, scale = 10 mm. Image E reproduced from Reiswig & Kelly (2018): 159, seafloor image 68, with permission from the *NIWA Biodiversity Memoir* Series editors. Images A, B, C, H captured by ROV Team GEOMAR, ROV *Kiel 6000* onboard RV *Sonne* (voyage SO254), courtesy of Project PoribacNewZ, GEOMAR, and ICBM.

**Figure 5:**
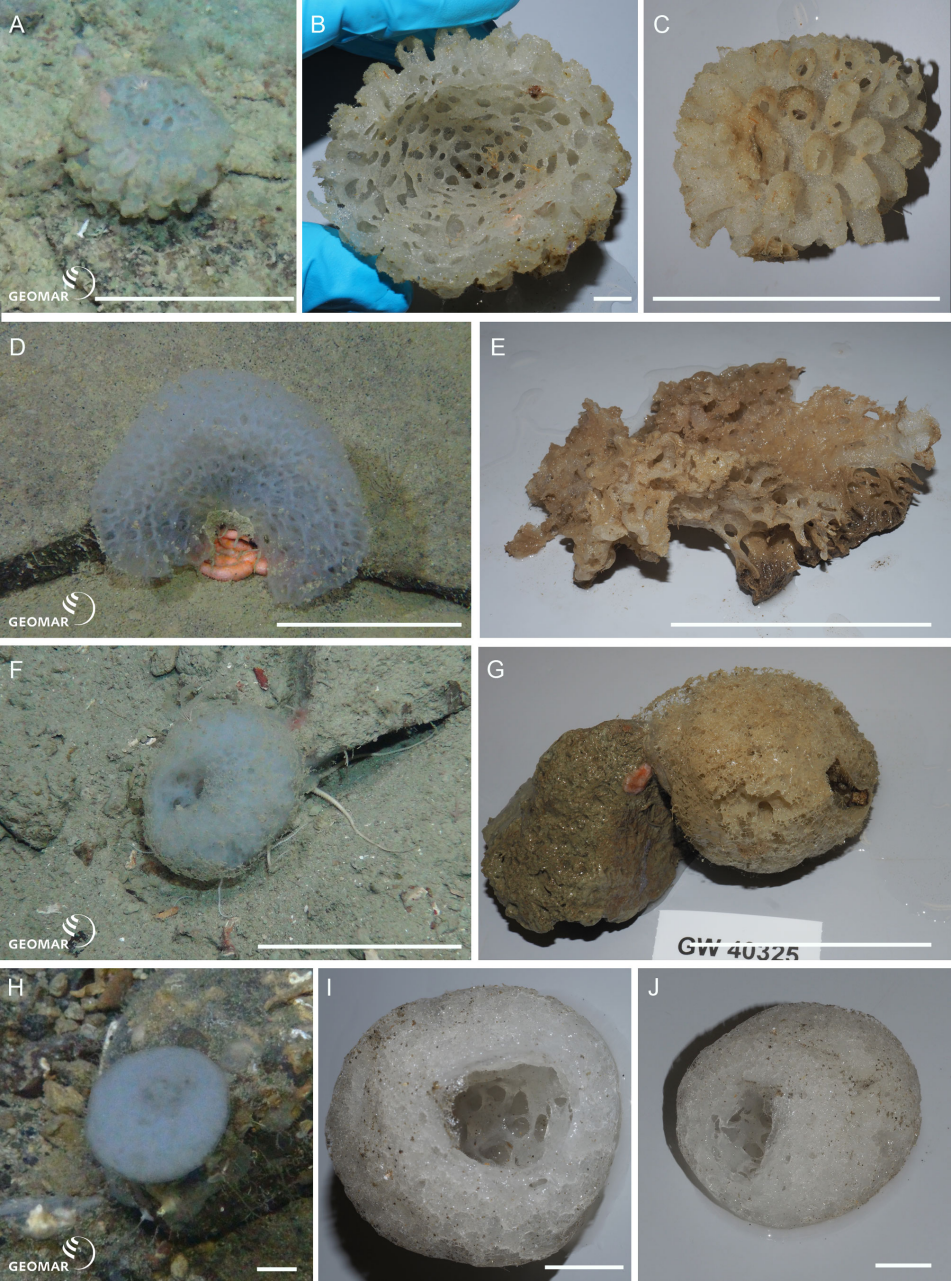
New species of Aulocalycidae and Leucopsacidae. *Aulocalyx* n. sp. (NIWA 126318) from Seamount 986, off Hawke Bay, eastern North Island, New Zealand, 875 m: (A) *In situ*, scale = 100 mm; (B) Deck image, apical view showing perforated interior, scale = 10 mm; (C) View of exterior, scale = 100 mm. *Rhabdodictyum* n. sp. (NIWA 126083) from the Kermadec Trench slope, north-eastern North Island, New Zealand, 4,833 m: (D) *In situ*, showing arched fan-shape, scale = 100 mm; (E) Deck image, scale = 100 mm. *Chaunoplectella* n. sp. (NIWA 126325) from Seamount 1247, off East Cape, eastern North Island, New Zealand, 1,389 m: (F) *In situ*, showing elongate ovoid body, scale = 100 mm; (G) Deck image, scale = 100 mm. *Leucopsacus* n. sp. (NIWA 126063) from West of Raoul Island, Kermadec Ridge, north-eastern North Island, New Zealand, 335 m: (H) *In situ*, showing cup-shaped body, scale = 10 mm; (I) (J) Deck images, scale = 10 mm. Images A, D, F, H captured by ROV Team GEOMAR, ROV *Kiel 6000* onboard RV *Sonne* (voyage SO254), courtesy of Project PoribacNewZ, GEOMAR, and ICBM.

**Figure 6:**
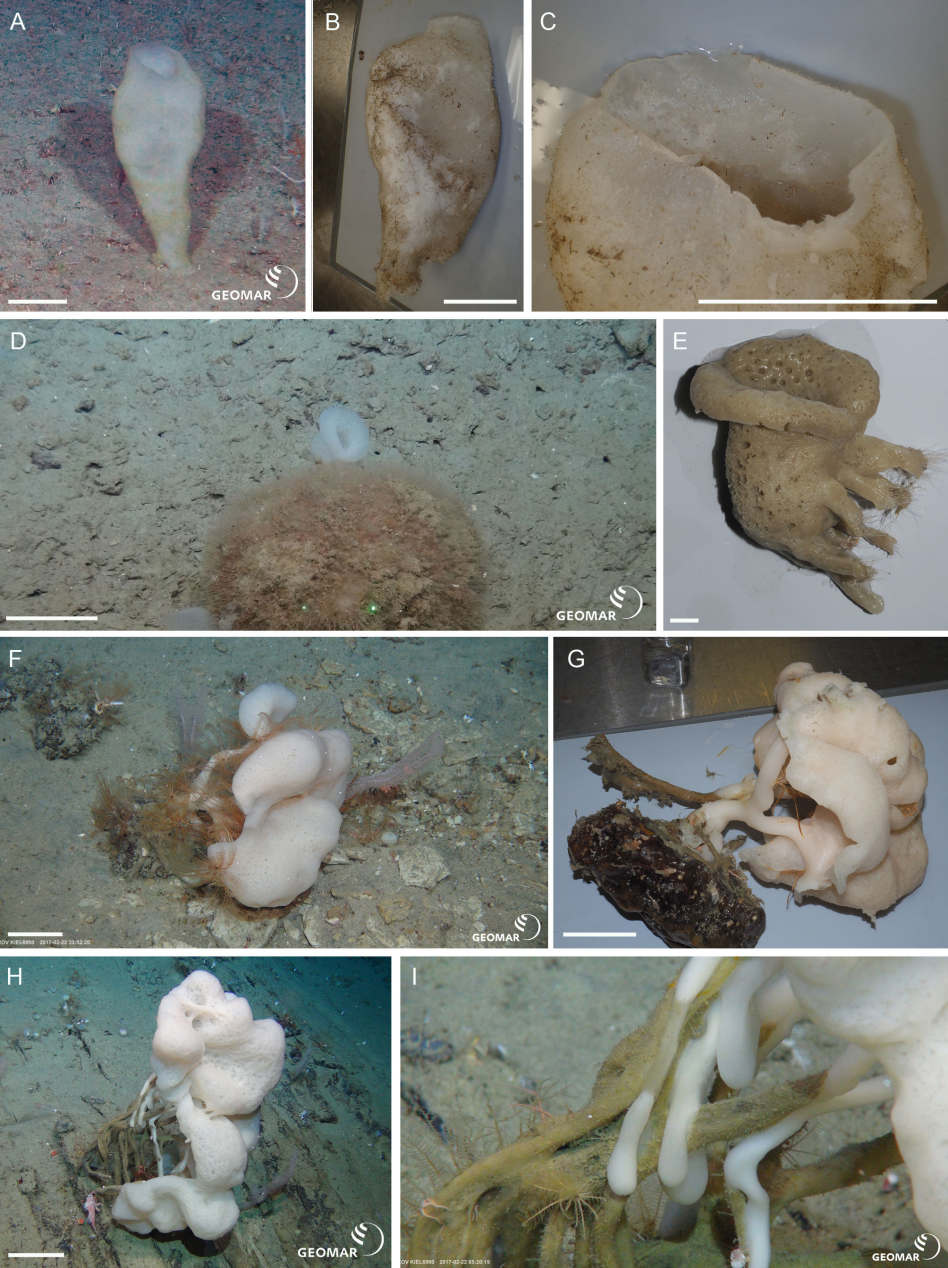
New species of Rossellidae. *Lophocalyx* n. sp. (NIWA 126005) from Kiwi Seamount, Three Kings Ridge, northern New Zealand, 760 m: (A) *In situ* image showing thin-walled, vase-shaped sponge, scale = 100 mm; (B) Deck image showing slightly collapsed body, scale = 100 mm; (C) thin upper body wall margin, scale = 100 mm. Lanuginellinae n. gen., n. sp. from Seamount 986, off Hawke Bay, eastern North Island, New Zealand, 802–893 m: (D) *In situ* image of chalice-shaped NIWA 126122, scale = 100 mm; (E) Deck image of NIWA 126122 showing leg-shaped attachment base, scale = 10 mm; (F) *In situ* image of NIWA 126310, scale = 100 mm; (G) Deck image of NIWA 126310, showing the hard trunk-shaped stem and broad, solid attachment base, scale = 100 mm; (H) *In situ* image of NIWA 126315, scale = 100 mm; (I) Close-up of NIWA 126315 *in situ* showing the striated, compressed hard basal stems from which the soft body arises. Images A, D, F, H, I captured by ROV Team GEOMAR, ROV *Kiel 6000* onboard RV *Sonne* (voyage SO254), courtesy of Project PoribacNewZ, GEOMAR, and ICBM.

Underwater (*in situ*) and deck photographs of the new species are provided in Figs. 2–6.

*Hyalonema* n. sp. (NIWA 126036) (Amphidiscosida: Hyalonematidae), abyssal basin between Three Kings and Colville Ridges, northern New Zealand, 4,158 m. Figures 2A–2C.

NIWA 126036 consists of an oval body, 36 mm high, on a slender, twisted ‘rope’ of six or more long, thick, anchoring spicules, the whole sponge rooted basally in sediment (A). The surface is smooth, felty, and appears faintly cross-hatched; the color *in situ* is white, and above water, tan (B, C). This specimen cannot presently be assigned to any existing subgenus of *Hyalonema*, and the material examined was too damaged to erect a new subgenus; for the time being this species is treated morphologically as “subgen. inc. sed”. Furthermore, the spiculation does not match any of the 15 species known from a 1,000 km radius around the collection site. Its most prominent spiculation feature is very long, whip-like (likely atrial) pinules.

*Pheronema* n. sp. (NIWA 126135, 126138) (Amphidiscosida: Pheronematidae), Southern Kermadec Ridge, northeastern North Island, New Zealand, 1,169 m. Figures 2D–2F.

NIWA 126135 and NIWA 126138 are barrel-shaped sponges, 105 mm, and 165 mm diameter, respectively (D), with a wide, smooth opening at the apex leading to a deep atrium that extends almost to the base of the sponge (E, F). The exterior is covered in a beard of very fine, hair-like spicules that trap sediment (F), the color *in situ* is pinkish brown, and above water, dark tan. These specimens clearly differ in spiculation from all 20 described species of the genus.

*Poliopogon* n. sp. (NIWA 126337) (Amphidiscosida: Pheronematidae), Southern Kermadec Ridge, northeastern North Island, New Zealand, 1,150 m. Figures 2G, 2H.

NIWA 126337 is a large pillar, about 400 mm high and 230 mm wide, with large concave inhalant and exhalent cones, attached to the substrate by a long, spreading fringe (G). The exterior surfaces are a smooth crosshatch and the margins of the aquiferous cones, thin. Color *in situ* is pale lemon tan, peach out of water (H). This specimen shows a unique shape and size range of microuncinates and shape and size of macramphidiscs, not known from any described species of this genus.

*Eurete* n. sp. 1 (NIWA 126028) (Sceptrulophora: Euretidae), abyssal basin between Three Kings and Colville Ridges, northern New Zealand, 4,160 m. Figures 3A–3C.

NIWA 126028 is a pedunculate, cavernous, honeycombed body, about 12 cm long and eight cm wide, arising from a tough, solid stem (A). The body is bulb-shaped with a restricted apex opening into a wide tubular, perforated atrium (B, C). Texture is crunchy, brittle, color *in situ* white, tan out of water. This specimen was identified as *Homoieurete* n. sp. 1 by HMR due to the presence of some discohexasters; however, this contradicts the molecular phylogenetic results (see below) and needs further investigation.

*Eurete* n. sp. 2 (NIWA 126276) (Sceptrulophora: Euretidae), Wairarapa Slope, southeastern North Island, New Zealand, 1,663 m. Figures 3D, 3E.

NIWA 126276 is a shallow chalice with corrugations visible as translucent and opaque lines radiating from the base of the sponge body in life (D), about eight cm diameter at the apex. The external sculpted corrugations are readily visible in the deck image (E). Color *in situ* is a faint pinkish white, tan out of water. This specimen was identified as *Homoieurete* n. sp. 2 by HMR due to presence of some discohexasters; however, this contradicts the molecular phylogenetic results (see below) and needs further investigation.

*Farrea occa* n. ssp. (NIWA 126307) (Sceptrulophora: Farreidae), Seamount 986, off Hawke Bay, eastern North Island, New Zealand, 893 m. Figures 3F, 3G.

NIWA 126307 is a thick, curved, cavernous, honeycombed fan, about seven cm wide, attached along the base (F). Texture is crunchy, brittle, color *in situ* white, tan out of water (G). This specimen has hooks on most of the anchorate clavules, which is a character not known from the ten described subspecies of *F. occa* (cf. Lopes, Hajdu & Reiswig, 2011: Table 2).

*Farrea* n. sp. (NIWA 126004) (Sceptrulophora: Farreidae), Seamount 986, off Hawke Bay, eastern North Island, New Zealand, 893 m. Figures 3H, 3I.

NIWA 126004 is a pedunculate, tree-shaped sponge with numerous, hollow, tubular branches arising from the primary stem, about 12 cm high, attached to hard substrate by a tough, solid base (H). The ends of the branches are flared and irregularly extended in places (I). Texture tough, crunchy, brittle, color *in situ* and out of water, white. This specimen has a combination of clavule types (pileate and anchorate with hooks), microscleres (oxyhexasters only), and morphology of surface pentactins (microtuberculated) that does not match with diagnoses of the 37 described accepted species given in Lopes, Hajdu & Reiswig (2011): Table 2) and later additions (Reiswig & Kelly, 2011; Reiswig & Stone, 2013; Reiswig, 2014; Reiswig, 2018; Reiswig, 2020; Boury-Esnault et al., 2017; Tabachnick et al., 2019).

*Corbitella* n. sp. (NIWA 126122, 126123, 126350) (Lyssacinosida: Euplectellidae), Southern Kermadec Ridge, northeastern North Island, New Zealand, 1,164–1,205 m. Figures 4A–4G.

NIWA 126122 (A, D), NIWA 126123 (B, E) and NIWA 126350 (C, F, G) have long, hispid, tubular bodies, up to about 400 mm long and about 100 mm wide, with a delicate corona of marginal spicules projecting in a broad, thin, wavy cuff on the upper body wall, with eyelash-like hexactine parietal prostalia projecting from the rim. These sponges usually harbour a pair of resident Venus shrimps. Reiswig & Kelly (2018: 84) assigned these three specimens to *Regadrella hispida* (Reiswig & Kelly, 2018), due to their apparent similarity to the holotype of *R*. *hispida*, illustrated in Reiswig & Kelly (2018): fig 31, F): all appeared to have an extremely hispid body wall and long marginal spicules projecting from the margin of the upper body wall. However, Reiswig & Kelly (2018) based their assignments to *R*. *hispida* on images only (NIWA 126122: seafloor image 68). Genetic differences in this study suggested these three specimens were a different species, and a new species of *Corbitella* rather than *Regadrella*. It is obvious to us now that *Corbitella* n. sp. has a thin, wavy, marginal cuff, that projects from the upper rim of the sponge body, whereas this appears to be absent in the, albeit damaged, holotype of *R*. *hispida*. Furthermore, the body of *R*. *hispida* is barrel-shaped, not tubular, and tall as in *Corbitella* n. sp.

*Trychella* n. sp. (NIWA 126306) (Lyssacinosida: Euplectellidae), Seamount 986, off Hawke Bay, eastern North Island, New Zealand, 893 m. Figures 4H, 4I.

NIWA 126306 is a thin-walled, bulbous, sack-shaped, sponge, 68 mm high (H), with a wide, globular, curved base, tapering to a broad, open apex surrounded by a thick margin from which project sparse, long, spine-like, marginal spicules (I). The wall is lumpy and punctate, texture slightly compressible, color *in situ* and out of water icy white. This specimen differs from the only described species, *T. kermadecensis*—among other spiculation characters—by having floricomes and lacking tetradiscs and pentadiscs.

*Aulocalyx* n. sp. (NIWA 126318, 126176, 126301) (Lyssacinosida: Aulocalycidae), Seamount 986, off Hawke Bay, eastern North Island, New Zealand, 875 m. Figures 5A–5C.

NIWA 126318 (A, B), NIWA 126176, and NIWA 126301, are cup-shaped sponges, about 85 mm wide, with a smooth, perforated interior (B) and heavily pocketed exterior (A, C). The tubular wall extensions are thin, delicate, and translucent, some are blind, and some are open-ended. The overall appearance is of a fragile, translucent flower. There are currently three described species in this genus: *A. irregularis* Schulze, 1886, *A. serialis* Dendy, 1916, and *A. australis* Reiswig & Kelly, 2011. The new specimens clearly differ in microsclere combination to these species: They have big spheric discohexasters, which are missing in *A. australis* and *A. irregularis*; have small spheric discohexasters, which are missing in *A. serialis*; lack big stellate discohexasters, which are present in *A. australis* and *A. serialis*; lack oxyhexactins, which are present in *A. australis* and *A. irregularis*; and have smaller rhopalasters than the described species.

*Rhabdodictyum* n. sp. (NIWA 126083) (Lyssacinosida: Aulocalycidae), Kermadec Trench slope, northeastern North Island, New Zealand, 4,833 m. Figures 5D, 5E.

NIWA 126083 is a moderately thin, beautifully regular, arching fan, about 15 cm wide, attached at the center of the base of the fan (D). The surface is punctate, the texture is compressible, appearing fibrous in close-up. Color *in situ* is translucent white and dark tan out of water (E). This specimen has a very different body shape than the only other known species, *R. delicatum* Schmidt, 1880 (cf. Reiswig, 2002b), and differs by having no other microscleres than spirodiscohexasters.

*Chaunoplectella* n. sp. (NIWA 126325) (Lyssacinosida: Leucopsacidae), Seamount 1247, off East Cape, eastern North Island, New Zealand, 1,389 m. Figures 5F, 5G.

NIWA 126325 is low, elongate to ovoid in overall form, about 85 mm diameter, with a large, deep, perforated atrium. The skeleton is very loose and airy with surface pentactins visible sitting on the surface. Color in life translucent icy white, tan out of water. This specimen lacks the large anchorate discohexasters typical for species of *Chaunoplectella* and could therefore be assigned to a new genus; however, given that it otherwise matches well with the diagnosis of *Chaunoplectella*, amendment of that diagnosis seems more appropriate.

*Leucopsacus* n. sp. (NIWA 126063) (Lyssacinosida: Leucopsacidae), West of Raoul Island, Kermadec Ridge, northeastern North Island, New Zealand, 335 m. Figures 5H–5J.

NIWA 126063 is cup-shaped with very thick walls and a deep atrium, about 45 mm high and 35 mm diameter (H, I). Surface relatively smooth with a relatively compact skeleton. Color in life translucent icy white, white out of water (I, J). This specimen clearly differs in spicule composition and dimensions from the five described species of the genus.

*Lophocalyx* n. sp. (NIWA 126005, 126014) (Lyssacinosida: Rossellidae: Lanuginellinae), Kiwi Seamount, Three Kings Ridge, northern New Zealand, 760 m. Figures 6A–6C.

NIWA 126005 and NIWA 126014 are sack-like sponges with a soft, felty, fibrous body that expands from a solid restricted attachment base, about 400 mm high (A), collapsing inwards slightly out of water (B). The upper body wall margin is thin and easily torn (C). Color *in situ* is white, whitish tan (attached detritus) out of water. This specimen clearly differs in spicule composition from the 12 described species of the genus.

Lanuginellinae n. gen. n. sp. (NIWA 126169, 126302, 126310, 126315, 126319) (Lyssacinosida: Rossellidae: Lanuginellinae), Seamount 986, off Hawke Bay, eastern North Island, New Zealand, 802–893 m. Figures 6D–6I.

NIWA 126169 (D, E) and other specimens examined are soft-bodied sponges up to 50 cm high, arising from solid, irregular, cylindrical, or laterally compressed stems of highly variable morphology. Stems are curved, anastomosing, or diverging to form branches which give rise to one or more soft, smooth, billowy, tulip to mushroom-shaped bodies, soft pinkish white *in situ* and out of water (D–I). This species has some similarities in spiculation to species of *Sympagella* Schmidt, 1870, and was initially suggested by HMR as a new species of that genus*.* However, the body shape is highly unusual and very distinct from the typical stalked, mushroom-like appearance of described *Sympagella* species. Furthermore, assignment to *Sympagella* would contradict the molecular evidence (see below). We therefore conclude that it is best classified in a new genus.

### Molecular phylogeny

**Figure 7:**
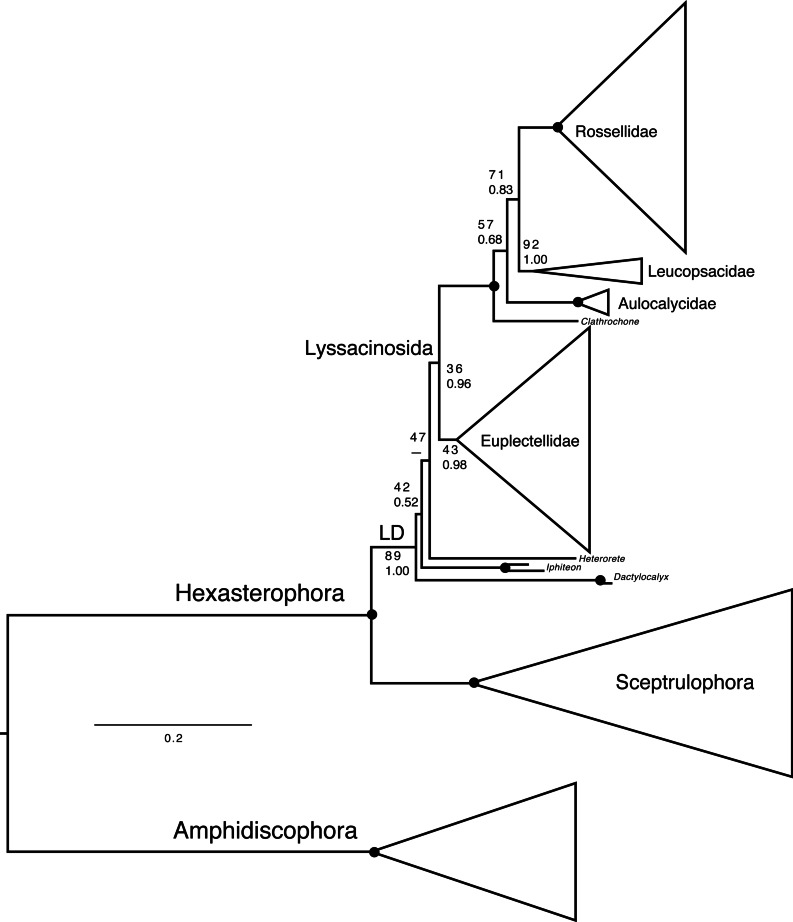
Expanded phylogeny of Hexactinellida based on combined 18S, 28S, 16S, and COI genes—overview of deep relationships. Phylogram is based on ML analysis; numbers at branches are rapid non-parametric bootstrap (Felsenstein, 1985; Stamatakis, Hoover & Rougemont, 2008) (BS; upper) and Bayesian posterior probability (PP; lower) values. BS values are based on 350 pseudoreplicates as determined by autoMRE bootstopping (Pattengale et al., 2010). –, clade not resolved in BI consensus tree. Nodes with black dot are fully supported by both methods (BS 100%, PP 1.00). LD, “LD clade” containing Lyssacinosida, Dactylocalycidae, and *Heterorete* (cf. Dohrmann et al., 2017). Scale bar, expected number of substitutions per site. For full phylogeny, see Fig. S2 (ML cladogram with BS and PP values), Fig. S3 (ML phylogram), and Fig. S4 (BI consensus cladogram with PP values).

Deep phylogenetic relationships of Hexactinellida (Fig. 7, Figs. S2–S4) are largely congruent with previous analyses (Dohrmann et al., 2017; Dohrmann, 2019). However, within Lyssacinosida, the sister group of Rossellidae is here Leucopsacidae with moderate bootstrap support (BS) and posterior probability (PP), instead of Aulocalycidae (Dohrmann, 2019) or a clade of Aulocalycidae + Leucopsacidae (Dohrmann et al., 2017: Fig. 7), in line with a genus-level total-evidence (DNA + morphology) analysis (Dohrmann et al., 2017: Fig. 10). Thus, the hypothesis that Aulocalycidae evolved their fused (dictyonal) skeleton from an ancestral leucopsacid-like choanosomal skeletal organization (Dohrmann et al., 2017: p. 17) could not be corroborated further by increased taxon sampling of these two small families.

Another discrepancy with Dohrmann (2019) concerns the low BS for monophyly of Lyssacinosida (36%), which was 82% in the previous study. However, we observed that after exclusion of the problematic *Heterorete* sp. (Hexasterophora *inc. sed.*), BS for Lyssacinosida rose to 75%, and when also removing the dactylocalycids *Dactylocalyx* and *Iphiteon*, even to 96% (Figs. S5–S6). Similarly, in the phylogeny including all taxa (Fig. 7, Fig. S2) BS for monophyly of Euplectellidae is only 43% but rose to 61% and 96%, respectively, under the reduced sampling schemes (Figs. S5–S6). These results indicate that *Heterorete* and the dactylocalycids behave as “rogue taxa” in the bootstrap analysis (see also Shen et al., 2019 for discussion of potential long-branch attraction issues). In contrast, BI was robust to these issues and yielded strong support for these well-established groups with the full dataset (Fig. 7, Figs. S2, S4). However, both methods were unable to resolve the exact positions of *Dactylocalyx*, *Iphiteon*, and *Heterorete*—clearly, more data are needed to determine their relationships (Dohrmann et al., 2017).

Below we briefly discuss the positions of the additional species for major hexactinellid subgroups (Amphidiscophora, Sceptrulophora, and the families of Lyssacinosida), and some implications for taxonomy and evolution.

**Figure 8:**
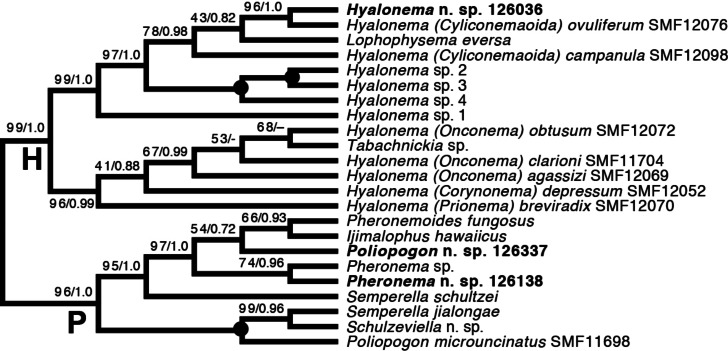
Expanded ML phylogeny of Hexactinellida—part Amphidiscophora. Taxa newly sequenced in this study are shown in bold font and with NIWA voucher number (126XXX). Taxa with SMF voucher numbers are from Kersken et al. (2018a). Numbers at branches are BS (left) and PP (right) values. –, clade not resolved in BI consensus tree (Fig. S4). Nodes with black dot are fully supported by both methods. H, Hyalonematidae; P, Pheronematidae.

*Amphidiscophora (*Fig. 8*)*.—Among Pheronematidae, monophyly of the type genus *Pheronema* is corroborated by inclusion of an additional species (*Pheronema* n. sp.). Inclusion of a second species of *Poliopogon* (*Poliopogon* n. sp.) reveals an apparent diphyly of this genus. However, the position of *Poliopogon* n. sp. is congruent with predictions of a total-evidence analysis (Dohrmann et al., 2017: Fig. 8), whereas the position of *P. microuncinatus* Kersken et al., 2018 close to *Schulzeviella* n. sp. might indicate generic misassignment by the describing authors (*i.e.,* Kersken, Janussen & Martínez Arbizu, 2018b). This gains some support from the presence in this species of the eponymous dagger-shaped microuncinates, which have also been described from *Schulzeviella gigas* (Schulze, 1886), originally placed in *Poliopogon* (see Tabachnick & Menshenina, 2002). A similar issue has been suggested for the apparent diphyly of *Semperella* (Dohrmann, 2019); additional investigations of the morphology of *P. microuncinatus* and *S. jialongae* Gong, Li & Qiu, 2015 will be necessary to resolve their taxonomy.

Among Hyalonematidae, *Hyalonema* n. sp. is very closely related to *H. (Cyliconemaoida) ovuliferum* Schulze, 1899, suggesting it should be placed in that subgenus. The placement of *Lophophysema* and *Tabachnickia* as ingroups of *Hyalonema* suggests that they might be better classified as subgenera of *Hyalonema*. As pointed out by Dohrmann et al. (2017) and Dohrmann (2019), major integrative taxonomic revisions of the family will be necessary to solve the phylogenetic status of subtaxa and their assignment to genera or subgenera. For further discussion of hyalonematid phylogeny, see Dohrmann (2019).

**Figure 9:**
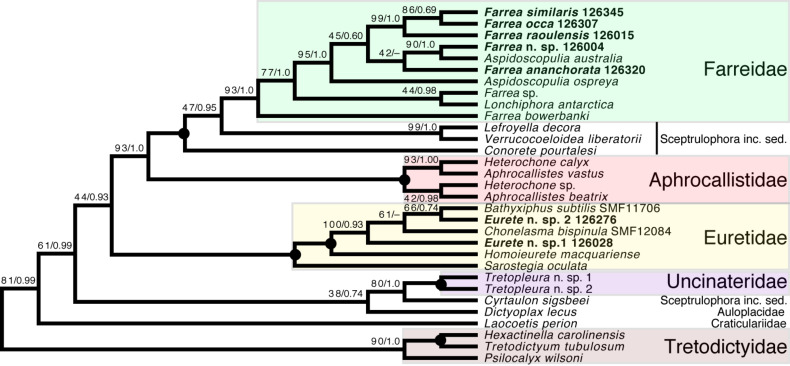
Expanded ML phylogeny of Hexactinellida—part Sceptrulophora. Taxa newly sequenced in this study are shown in bold font and with NIWA voucher number (126XXX). Taxa with SMF voucher numbers are from Kersken et al. (2018a). Numbers at branches are BS (left) and PP (right) values. –, clade not resolved in BI consensus tree (Fig. S4). Nodes with black dot are fully supported by both methods. inc. sed., *incertae sedis*.

*Sceptrulophora (*Fig. 9*)*—Addition of five further species of *Farrea* corroborates the artificial nature of the type genus of Farreidae (see discussion in Dohrmann, 2019) but also reveals that the genus *Aspidoscopulia* is not monophyletic. This implies that aspidoscopules (the diagnostic spicule type of *Aspidoscopulia*) are a poor phylogenetic character that can be expressed in multiple unrelated species. Therefore, the two genera—as well as *Lonchiphora* (two species), which is also nested within *Farrea* (Fig. 9; Dohrmann, 2019)—should be considered to be synonymized in future revisions of the family.

Regarding the “waste-bin” family Euretidae (Reiswig & Dohrmann, 2014; Dohrmann et al., 2017), we here provide the first sequences of the type genus *Eurete* (*Eurete* n. sp. 1 and 2). Both are firmly placed in the *Homoieurete*-*Sarostegia*-*Chonelasma*-*Bathyxiphus* clade (Kersken et al., 2018a), which should therefore become the new core for a revised scope of Euretidae (cf. Dohrmann, 2019). Consequently, we move *Homoieurete* and *Sarostegia* from Sceptrulophora *inc. sed.* (Reiswig & Dohrmann, 2014; Dohrmann et al., 2017) back to Euretidae, and instead transfer *Conorete*, *Lefroyella*, and *Verrucocoeloidea* to Sceptrulophora *inc. sed.* The position of *Eurete* within Sceptrulophora recovered here contrasts with a previous total-evidence analysis (Dohrmann et al., 2017: Fig. 9), although analysis of morphological data alone indicated a closer relationship with *Chonelasma* (Dohrmann et al., 2017: Fig. 5). *Eurete* itself is here recovered paraphyletic, with *Eurete* n. sp. 2 more closely related to *Bathyxiphus subtilis* Schulze, 1899 and *Chonelasma bispinula* Kersken, Janussen & Martínez Arbizu, 2019 (although with poor support). However, this result must be taken with caution since the latter species are only represented by a 16S sequence (*B. subtilis*) and a partial 18S sequence (*C. bispinula*), respectively (Kersken et al., 2018a), which could have disturbed accurate reconstruction of this quartet due to excessive missing data; when the incompletely sampled *B. subtilis* and *C. bispinula* are excluded, *Eurete* is recovered as monophyletic (*e.g.*, Fig. S1). Despite the poor resolution of the relationships of *Bathyxiphus*, *Chonelasma*, and NIWA 126028 and 126276, they nonetheless group together in a strongly supported clade as sister to *Homoieurete macquariense* Reiswig & Kelly, 2011, separated by a relatively long branch (Fig. 9, Fig. S3). Thus, assignment of the new species to *Homoieurete* due to presence of discohexasters (see above) seems inappropriate; instead, generic diagnoses of *Homoieurete* and *Eurete* should be emended to better distinguish them morphologically.

**Figure 10:**
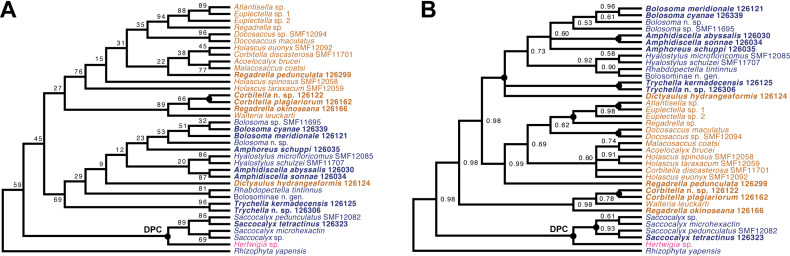
Expanded ML (A) and BI (B) phylogenies of Hexactinellida –part Euplectellidae. Taxa newly sequenced in this study are shown in bold font and with NIWA voucher number (126XXX). Taxa with SMF voucher numbers are from Kersken et al. (2018a). *Saccocalyx microhexactin* sequences were concatenated from Kersken et al. (2018a) (18S, 28S) and Gong, Li & Qiu (2015) (16S). *Rhizophyta yapensis* sequences are from Shen et al. (2019). Taxa are color-coded according to body shape (see text). Brown: venus-flower basket type or similar; blue: mushroom/wine glass (bolosomine) type; pink: other (plexiform walls composed of dichotomously branching-anastomosing tubes; cf. (Tabachnick, 2002a)). DPC, “discoplumicome-clade” of Dohrmann et al. (2017). (A) ML tree. Numbers at branches are BS values. Nodes with black dot are fully supported (BS 100%). (B) BI tree. Numbers at branches are PP values. Nodes with black dot are fully supported (PP 1.00).

*Lyssacinosida: Euplectellidae (*Fig. 10*)*—With 13 so far unsequenced species, most of them recently described or new to science (Reiswig & Kelly, 2018; this study), in eight genera (four sequenced here for the first time), Euplectellidae was the best-represented family in the *Sonne* SO254 collection (Table 1; Table S1). As in Shen et al. (2019), *Rhizophyta yapensis* and the discoplumicome-bearing clade (DPC, here represented by *Hertwigia* and *Saccocalyx*; cf. Dohrmann et al., 2017) form successive sister groups to the remaining euplectellids in both the ML (Fig. 10A) and BI (Fig. 10B) trees. Notably, regarding the deeper relationships of the latter, there were marked discrepancies between results of the two methods.

In general, monophyly of *Trychella*, *Amphidiscella*, *Saccocalyx*, and *Bolosoma* could be (further) corroborated here. In contrast, *Regadrella* and *Corbitella* are clearly recovered as polyphyletic, indicating that these relatively poorly defined genera are in need of revision. *Amphidiscella* and the two recently described new genera of Bolosominae (*Amphoreus* and *Trychella*; Reiswig & Kelly, 2018) expectedly group with the remainder of that subfamily (except for *Saccocalyx* and *Rhizophyta*; see Dohrmann et al., 2017; Shen et al., 2019). However, this clade also includes the newly sequenced *Dictyaulus hydrangeaformis*, which has a typical venus-flower basket (VFB) body shape (Reiswig & Kelly, 2018), in contrast to the mushroom- or wine glass-shaped bolosomins. Further, in the BI tree, a clade containing members of the VFB genera *Corbitella* and *Regadrella* is strongly supported as the sister group to a clade containing the majority of VFB and bolosomine species. Thus, the “Bolosominae *sensu stricto*” and “VFB clade” concepts proposed by Dohrmann et al. (2017) as a starting point for a new subfamily classification of Euplectellidae could not be further corroborated here (see also Shen et al., 2019). A natural subgrouping of euplectellid genera therefore seems out of reach and should best be abandoned.

**Figure 11:**
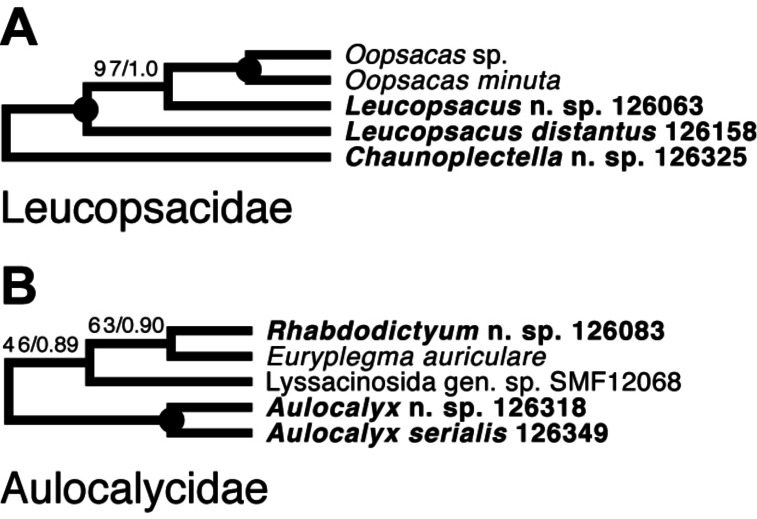
Expanded ML phylogeny of Hexactinellida –part Leucopsacidae (A) and Aulocalycidae (B). Taxa newly sequenced in this study are shown in bold font and with NIWA voucher number (126XXX). Taxon with SMF voucher number is from Kersken et al. (2018a). Numbers at branches are BS (left) and PP (right) values. Nodes with black dot are fully supported by both methods.

*Lyssacinosida: Leucopsacidae and Aulocalycidae (*Fig. 11*)*—Monophyly of these two small and thus far poorly sampled families is further supported here by inclusion of *Chaunoplectella* and *Leucopsacus* (Leucopsacidae), and *Rhabdodictyum* and *Aulocalyx* (Aulocalycidae), respectively. Genus-level taxon sampling is thus complete for Leucopsacidae; Aulocalycidae contains four further genera, but it is unclear if the elusive lyssacinosid specimen SMF12068 (cf. Dohrmann, 2019) belongs to any of those or represents a new genus. Among Aulocalycidae, monophyly of *Aulocalyx* is confirmed, whereas among Leucopsacidae the type genus appears paraphyletic with strong support, as it includes *Oopsacas*. This latter result is somewhat surprising as *Leucopsacus* is morphologically well defined (Tabachnick, 2002b); clearly this needs to be investigated further before any taxonomic action can be taken. A possible reason could be gene-tree-species-tree conflict as the genus was resolved as monophyletic (81% BS) in the 18S single-gene tree (not shown).

**Figure 12:**
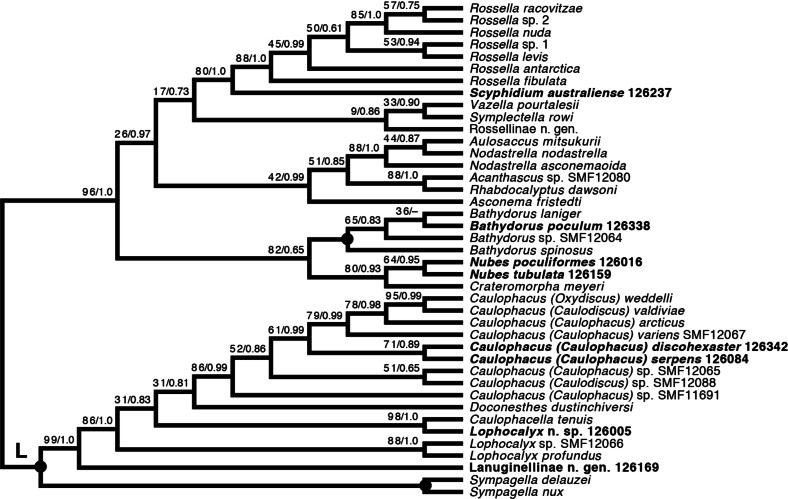
Expanded ML phylogeny of Hexactinellida –part Rossellidae. Taxa newly sequenced in this study are shown in bold font and with NIWA voucher number (126XXX). Taxa with SMF voucher numbers are from Kersken et al. (2018a). 28S, 16S, and COI of *Rossella* sp. 2, *R. antarctica*, *R. levis*, and *R. fibulata*, and COI of *R. racovitzae* are from Vargas et al. (2017) (GD4075, SMF11734, SMF11728, SMF11732, SMF11733). Numbers at branches are BS (left) and PP (right) values. –, clade not resolved in BI consensus tree (Fig. S4). Nodes with black dot are fully supported by both methods. L, Lanuginellinae.

*Lyssacinosida: Rossellidae (*Fig. 12*)*—Monophyly of *Caulophacus* and non-monophyly of its subgenera *Caulophacus* and *Caulodiscus* (Kersken et al., 2018a; Dohrmann, 2019) is further supported here by inclusion of *C. (Caulophacus) serpens* (described in Reiswig et al., 2021) and *C. (Caulophacus) discohexaster* (re-described in Reiswig et al., 2021), which seem to be closely related. Also, according to the 16S tree (Fig. S1; see comment in section *“Specimen collection and identification”*), *C. (Caulophacus) ramosus* (NIWA126085; described in Reiswig et al., 2021) appears to be very closely related to *C. (Caulophacus) arcticus*, *C. (Caulodiscus) valdiviae* Schulze, 1904, and *C. (Oxydiscus) weddelli* Janussen, Tabachnick & Tendal, 2004, although it shows no obvious morphological similarities to these species, beyond the shared characteristics of the genus. Inclusion of another species of *Lophocalyx*, *Lophocalyx* n. sp., did not corroborate the monophyly of this genus, as the species seems to be close to *Caulophacella*. More taxonomic work on the morphology of the poorly known *Caulophacella* and its relationship to *Lophocalyx*, as well as better taxon sampling of the latter (currently 12 described species) will be necessary to settle this issue. In any case, *Caulophacella* clearly groups outside *Caulophacus*, confirming its status as a separate genus (Gong et al., 2023; *contra* Boury-Esnault et al., 2015). The other species (and new genus) of Lanuginellinae sampled during *Sonne* Cruise SO254 with several specimens (NIWA 126169 and four other samples; Table 1), despite having some similarities in spiculation to *Sympagella* (see above), the sister taxon to all remaining lanuginellines, clearly diverges after that genus in the molecular phylogeny and forms a distinct branch in the Lanuginellinae clade (Fig. 12, Fig. S3).

Among the remainder of Rossellidae, the recently described genus *Nubes* (Reiswig et al., 2021) is monophyletic and appears closely related to *Crateromorpha*. Although the two genera are overall similar in spiculation, no striking potential synapomorphies are apparent to us, except perhaps the presence of a peduncle in *Crateromorpha* and *N. poculiformis*, which is however a homoplasy-prone character. Monophyly of *Bathydorus*, reconstructed here as the sister group of *Crateromorpha* + *Nubes*, is further confirmed by inclusion of *B. poculum* (see Reiswig et al., 2021 for description). *Scyphidium australiense* is strongly supported as the sister group of *Rossella*. Although the taxonomic history of the two genera is somewhat intermingled (see Tabachnick, 2002c), again no strong support from morphological characters is apparent to us. It is very closely related to *S. variospinosum* (see Reiswig et al., 2021 for description) according to the 16S analysis (Fig. S1). In fact, the sequences of the three investigated *Scyphidium* specimens (Reiswig et al., 2021) are so similar that, based on the molecular results only, we assumed they were conspecific (hence we did not attempt to sequence the other markers for all of them). This high genetic similarity likely points to a very recent divergence or incipient speciation of *S. variospinosum*.

### Chemical fingerprinting

**Figure 13:**
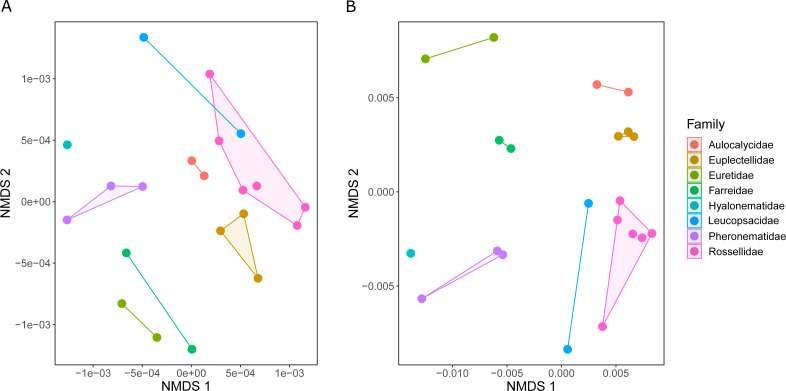
Similarity (NMDS based on Euclidean distances) of the metabolic profiles of different New Zealand glass sponges. (A) All metabolites with intensities > 10,000 (4,742 compounds). (B) Only major compounds (intensities > 5,000,000, 112 compounds).

**Figure 14:**
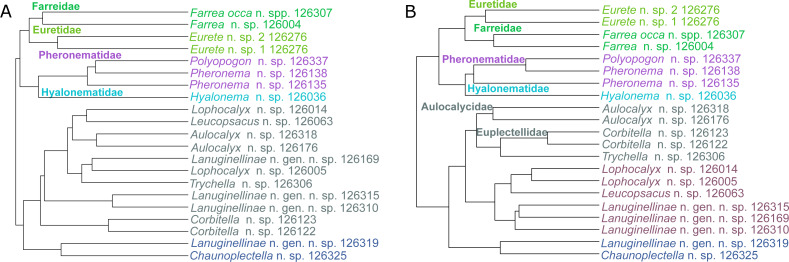
Hierarchical cluster (average algorithm) of the metabolic profiles of different New Zealand glass sponges. Different colors indicate identified significant clusters (KGS penalty). (A) All metabolites with intensities > 10,000 (4,742 compounds). (B) Only major compounds (intensities > 5,000,000, 112 compounds).

The metabolic profiles of different glass sponge families were significantly different (ANOSIM *R* = 0.75, Fig. 13A); however, these differences were stronger when only the major metabolites were analyzed (ANOSIM *R* = 0.87, *p* = 0.001, Fig. 13B). When all metabolites were analyzed, five significantly different clusters were obtained (Fig. 14A). The cluster analysis was congruent with the NMDS (separation along NMDS1) and showed a first separation into two major clusters: cluster 1, which contained the families Farreidae, Euretidae, Pheronematidae and Hyalonematidae, and cluster 2, which contained the families Euplectellidae, Aulocalycidae, Leucopsacidae, and Rossellidae. While cluster 2 is congruent with monophyly of Lyssacinosida, cluster 1 contradicts monophyly of Hexasterophora (*i.e.,* Sceptrulophora [here Farreidae + Euretidae] closer to Amphidiscophora [Pheronematidae + Hyalonematidae] than Lyssacinosida), suggesting limitations of chemical fingerprinting at this deep phylogenetic level. The first cluster split into two subclusters separating the two Amphidiscophora families (which formed by themselves unique clusters) from the Farreidae and Euretidae, which were also clearly separated into statistically different clusters (Fig. 14A). Metabolic fingerprinting of all metabolites did not allow a clear resolution of cluster 2, *i.e.,* of the four families of Lyssacinosida, only Aulocalycidae was recovered. Analysis of only major metabolites allowed identification of 7 statistically different clusters (Fig. 14B), with Euplectellidae now also being clearly separated. However, genera of Rossellidae and Leucopsacidae were still intermingled. One reason for the unclear taxonomic resolution when all metabolites were included could be the time span between actual collection at depth and preservation of the sponge pieces on board. This could have led to the production of some additional minor metabolites by the sponge as a result of the physical injury during collection and transport to the surface and ship (which could have been up to 6 h for certain specimens). Despite this, in general a good clustering by family and genus can be observed, suggesting a higher replication could allow a better chemotaxonomic discrimination (possibly to genus or species level).

## Conclusions & Outlook

This study has further demonstrated that the deep waters surrounding New Zealand are a biodiversity hotspot for glass sponges (see Reiswig et al., 2021). Known diversity of NZ hexactinellids was increased by 15 species and four genera, including 14 species and one genus new to science. Furthermore, the material collected on *Sonne* cruise SO254 allowed us to increase the taxonomic sampling of Hexactinellida by 37 species and 12 genera (compared to Kersken et al., 2018a; Dohrmann, 2019), providing new insights into the systematics and evolution of this fascinating group of sponges.

The use of untargeted metabolomics as a chemotaxonomic tool (*i.e.,* phylometabolomics) to complement morphological and molecular tools in systematics analyses has been previously evidenced in tropical zoanthids (Jaramillo et al., 2018). Although the potential of this novel tool in sponge systematics has been previously discussed (Boury-Esnault et al., 2013; Galitz et al., 2021; Li, Kelly & Tasdemir, 2021), this is the first study to demonstrate the applicability of phylometabolomics in a divergent class of sponges, the Hexactinellida. The clustering based on metabolomic fingerprinting, which shows some promising congruence with the systematics and phylogeny of glass sponges, shows that this method could become an additional taxonomic tool for this group. Although some incongruencies with the molecular phylogeny remained in this pilot study, an increased taxonomic sampling could allow for a clearer resolution in the future.

Largely through the work of Kersken et al. (2018a) and this study, taxon sampling for molecular phylogenetics of glass sponges with the markers established by Dohrmann et al. (2008) and Dohrmann et al. (2012) has more than doubled since the last class-wide study (Dohrmann et al., 2017; from 73 to 148 species). However, several issues still remain that should be addressed in future, more targeted projects. Most importantly, despite some attempts (MD, unpubl.), the phylogenetic position of Lychniscosida Schrammen, 1903, a paleontologically very important relict group (Krautter, 2002), could not be determined yet with molecular analyses, so the morphology-based hypothesis that this dictyonal order is closer to Lyssacinosida than to Sceptrulophora (Mehl, 1992; Dohrmann et al., 2017) remains to be tested. More generally, the branching order at the base of the “LD clade” needs to be better resolved to determine the exact sister group of Lyssacinosida and confirm or reject monophyly of Dactylocalycidae (see Dohrmann et al., 2017). Also, several monotypic families and other important genera still await to be sampled (*e.g.*, Monorhaphididae/*Monorhaphis*, Cribrospongiidae/*Stereochlamis*, Fieldingiidae/*Fieldingia*, *Auloplax* [Auloplacidae], *Hyaloplacoida* [Lyssacinosida *inc. sed.* ], *Myliusia* [Hexasterophora *inc. sed.* ]). Denser sampling is further required to resolve internal relationships of the larger families Hyalonematidae, Euplectellidae, and Rossellidae, and to achieve a natural classification of the numerous genera of “Euretidae”. Finally, the apparent or possible para- or polyphyly of some genera needs to be further investigated and resolved with integrative taxonomic approaches (*e.g.*, *Hyalonema*, *Semperella*, *Farrea*, *Euplectella*, *Corbitella*, *Holascus*, *Leucopsacus*, *Lophocalyx*, *Nodastrella*). Last but not least, genomic or transcriptomic datasets of additional glass sponge species could help to further test the deeper relationships of the class and provide important insights into its evolution.

##  Supplemental Information

10.7717/peerj.15017/supp-1 Supplemental Information 1 Aligned DNA sequences before trimming

10.7717/peerj.15017/supp-2 Supplemental Information 2 Sampling data of all glass sponge specimens collected during RV *Sonne* cruise SO254

10.7717/peerj.15017/supp-3 Supplemental Information 3 Maximum-likelihood phylogeny of glass sponges based on the 16S alignment of Dohrmann et al. (2017) plus all successfully sequenced SONNE SO254 specimens (displayed in bold font as NIWA voucher number [126XXX] followed by abbreviated family name) The tree was inferred with RAxML (Stamatakis, 2014) using the “-f a” option. Numbers at branches are rapid non-parametric bootstrap values (Felsenstein, 1985; Stamatakis, Hoover & Rougemont, 2008) based on 600 pseudoreplicates as determined by autoMRE bootstopping (Pattengale et al., 2010). Scale bar, expected number of substitutions per site.

10.7717/peerj.15017/supp-4 Supplemental Information 4 Full expanded phylogeny of Hexactinellida based on ML analysis of combined 18S, 28S, 16S, and COI genes For better readability, the tree is displayed as a cladogram; for branch lengths see Fig. S3. Taxa newly sequenced in this study are shown in bold font and with NIWA voucher number (126XXX). Taxa with SMF (Senckenberg Museum, Frankfurt, Germany) voucher numbers are from Kersken et al. (2018a). *Saccocalyx microhexactin* sequences were concatenated from Kersken et al. (2018a) (18S, 28S) and Gong, Li & Qiu (2015) (16S). *Rhizophyta yapensis* sequences are from Shen et al. (2019). 28S, 16S, and COI of *Rossella* sp. 2, *R. antarctica*, *R. levis*, and *R. fibulata*, and COI of *R. racovitzae* are from Vargas et al. (2017) (GD4075, SMF11734, SMF11728, SMF11732, SMF11733). Numbers at branches are rapid non-parametric bootstrap (Felsenstein, 1985; Stamatakis, Hoover & Rougemont, 2008) (BS; left) and Bayesian posterior probability (PP; right) values. BS values are based on 350 pseudoreplicates as determined by autoMRE bootstopping (Pattengale et al., 2010). –, clade not resolved in BI consensus tree. *, clade contradicted by BI consensus tree (cf. Fig. S4). inc. sed., *incertae sedis*; L, Lanuginellinae; LD, “LD clade” of Dohrmann et al. (2017).

10.7717/peerj.15017/supp-5 Supplemental Information 5 Phylogram version of the tree shown in Fig. S2. Scale bar, expected number of substitutions per site.

10.7717/peerj.15017/supp-6 Supplemental Information 6 Phylogeny of Hexactinellida estimated by BI analysis 50% majority-rule consensus cladogram. PP values ¡ 1.00 shown at nodes.

10.7717/peerj.15017/supp-7 Supplemental Information 7 Phylogeny of Hexactinellida estimated by ML analysis excluding *Heterorete* BS values for crown Lyssacinosida and crown Euplectellidae highlighted.

10.7717/peerj.15017/supp-8 Supplemental Information 8 Phylogeny of Hexactinellida estimated by ML analysis excluding *Heterorete* and Dactylocalycidae BS values for crown Lyssacinosida and crown Euplectellidae highlighted.

## Additional Information and Declarations

### Field Study Permissions

The following information was supplied relating to field study approvals (i.e., approving body and any reference numbers): Permit to collect marine organisms was provided by the Ministry of Foreign Affairs and Trade, Wellington, New Zealand.

### Data Availability

The following information was supplied regarding data availability: The sequences are available at the European Nucleotide Archive (ENA; OX394254-OX394279, OX400493-OX400575, OX400593-OX400660, and OQ301566 is available at GenBank.
