# Supplementary figures and images for: Expanded sampling of New Zealand glass sponges (Porifera: Hexactinellida) provides new insights into biodiversity, chemodiversity, and phylogeny of the class
Source: PeerJ. 2023 Apr 27;11:e15017. doi: 10.7717/peerj.15017 (PMC10149058; doi:10.7717/peerj.15017)

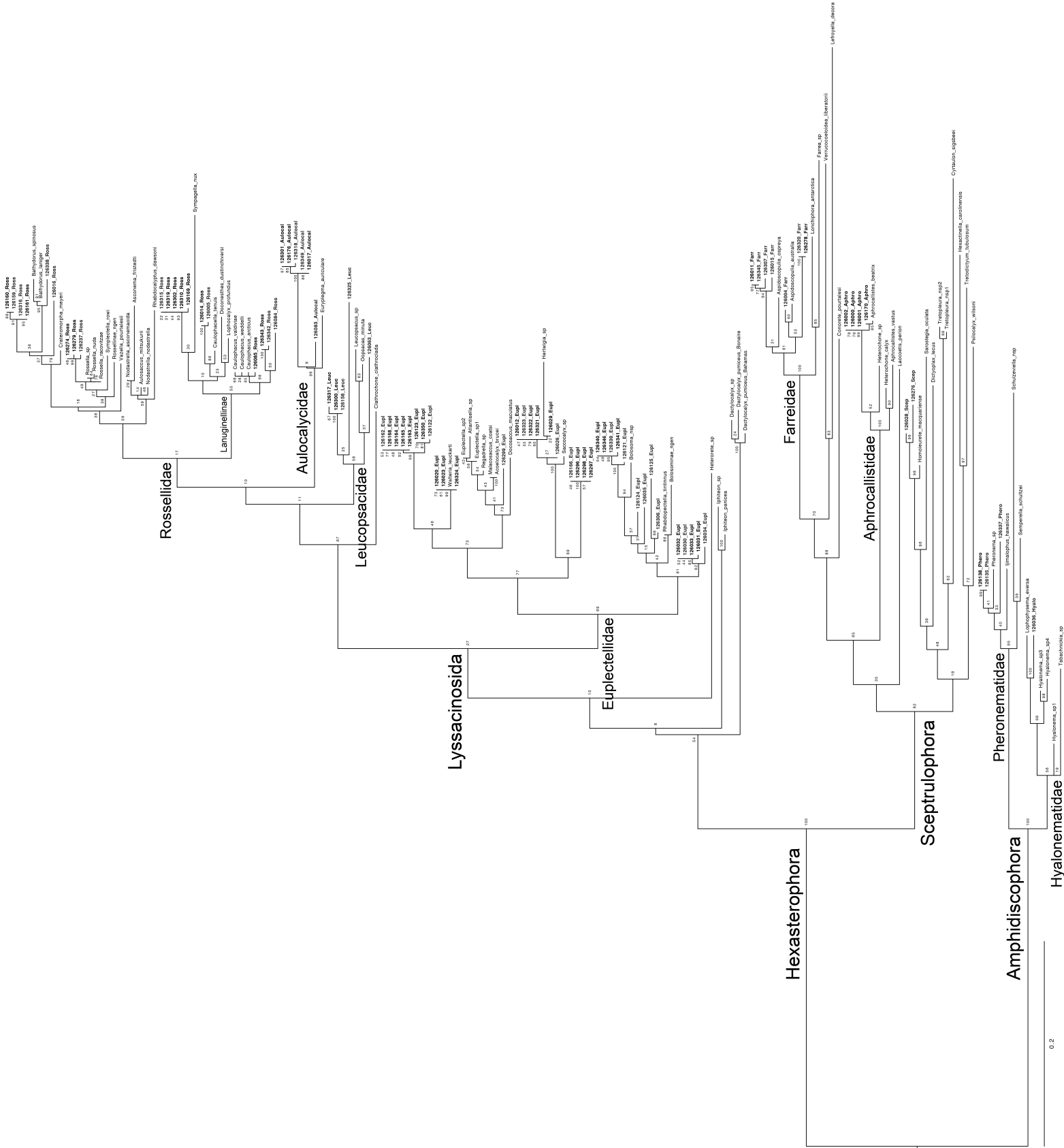

Supplement: Supplemental Information 3 — The tree was inferred with RAxML (Stamatakis, 2014) using the “-f a” option. Numbers at branches are rapid non-parametric bootstrap values (Felsenstein, 1985; Stamatakis, Hoover & Rougemont, 2008) based on 600 pseudoreplicates as determined by autoMRE bootstopping (Pattengale et al., 2010). Scale bar, expected number of substitutions per site. [file peerj-11-15017-s003.pdf]

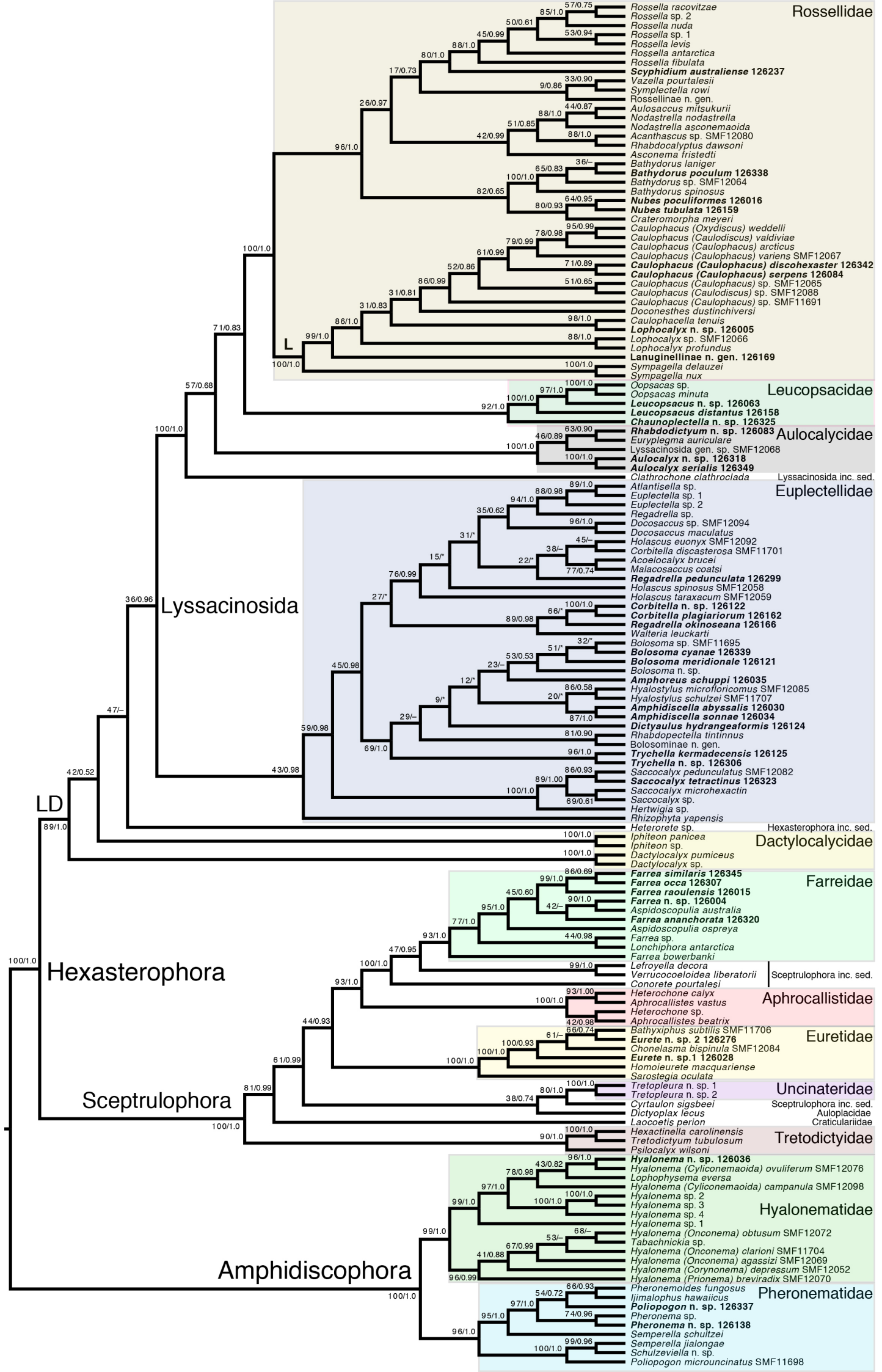

Supplement: Supplemental Information 4 — For better readability, the tree is displayed as a cladogram; for branch lengths see Fig. S3. Taxa newly sequenced in this study are shown in bold font and with NIWA voucher number (126XXX). Taxa with SMF (Senckenberg Museum, Frankfurt, Germany) voucher numbers are from Kersken et al. (2018a). Saccocalyx microhexactin sequences were concatenated from Kersken et al. (2018a) (18S, 28S) and Gong, Li & Qiu (2015) (16S). Rhizophyta yapensis sequences are from Shen et al. (2019). 28S, 16S, and COI of Rossella sp. 2, R. antarctica, R. levis, and R. fibulata, and COI of R. racovitzae are from Vargas et al. (2017) (GD4075, SMF11734, SMF11728, SMF11732, SMF11733). Numbers at branches are rapid non-parametric bootstrap (Felsenstein, 1985; Stamatakis, Hoover & Rougemont, 2008) (BS; left) and Bayesian posterior probability (PP; right) values. BS values are based on 350 pseudoreplicates as determined by autoMRE bootstopping (Pattengale et al., 2010). –, clade not resolved in BI consensus tree. *, clade contradicted by BI consensus tree (cf. Fig. S4). inc. sed., incertae sedis; L, Lanuginellinae; LD, “LD clade” of Dohrmann et al. (2017). [file peerj-11-15017-s004.pdf]

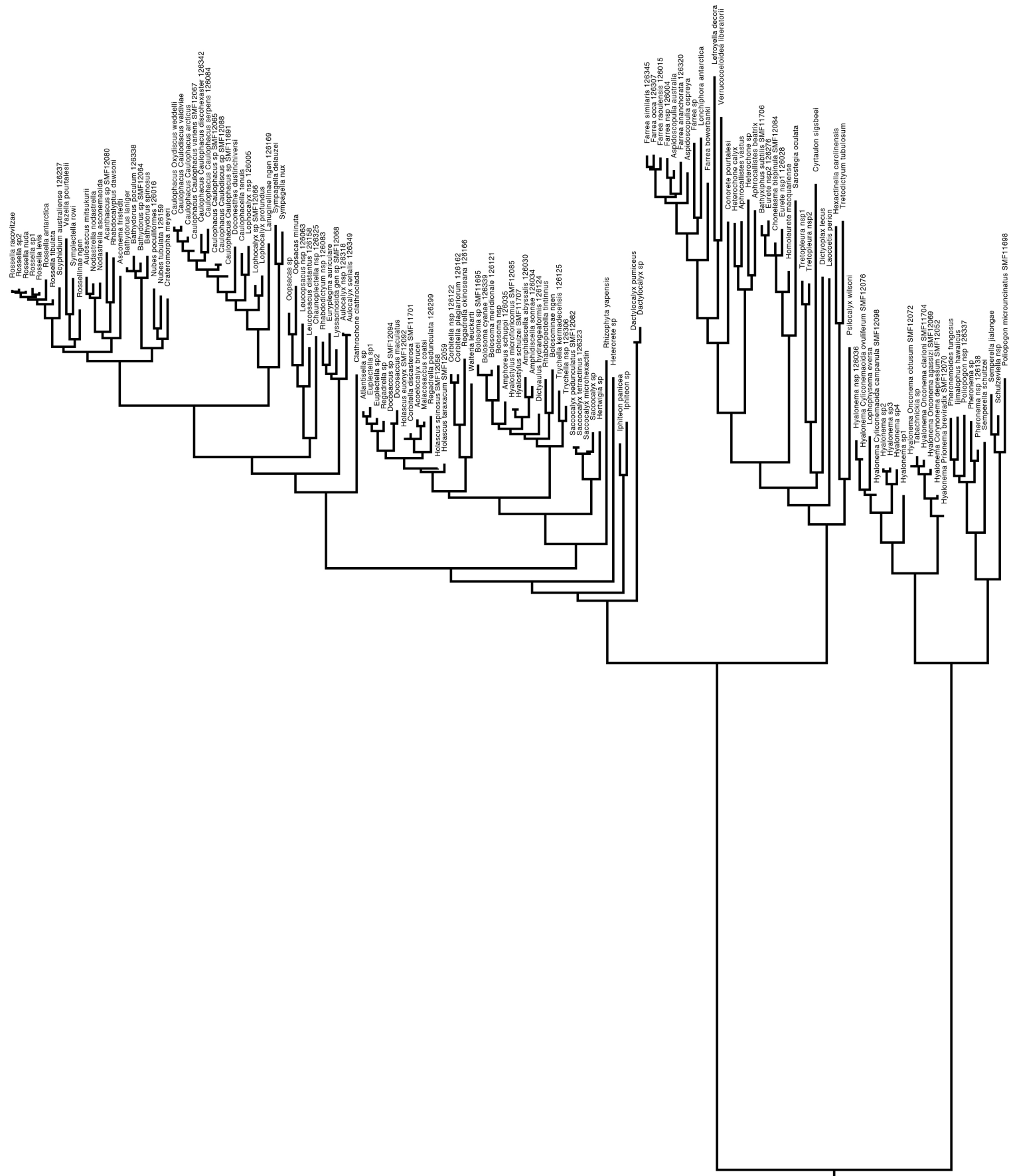

Supplement: Supplemental Information 5 — Scale bar, expected number of substitutions per site. [file peerj-11-15017-s005.pdf]

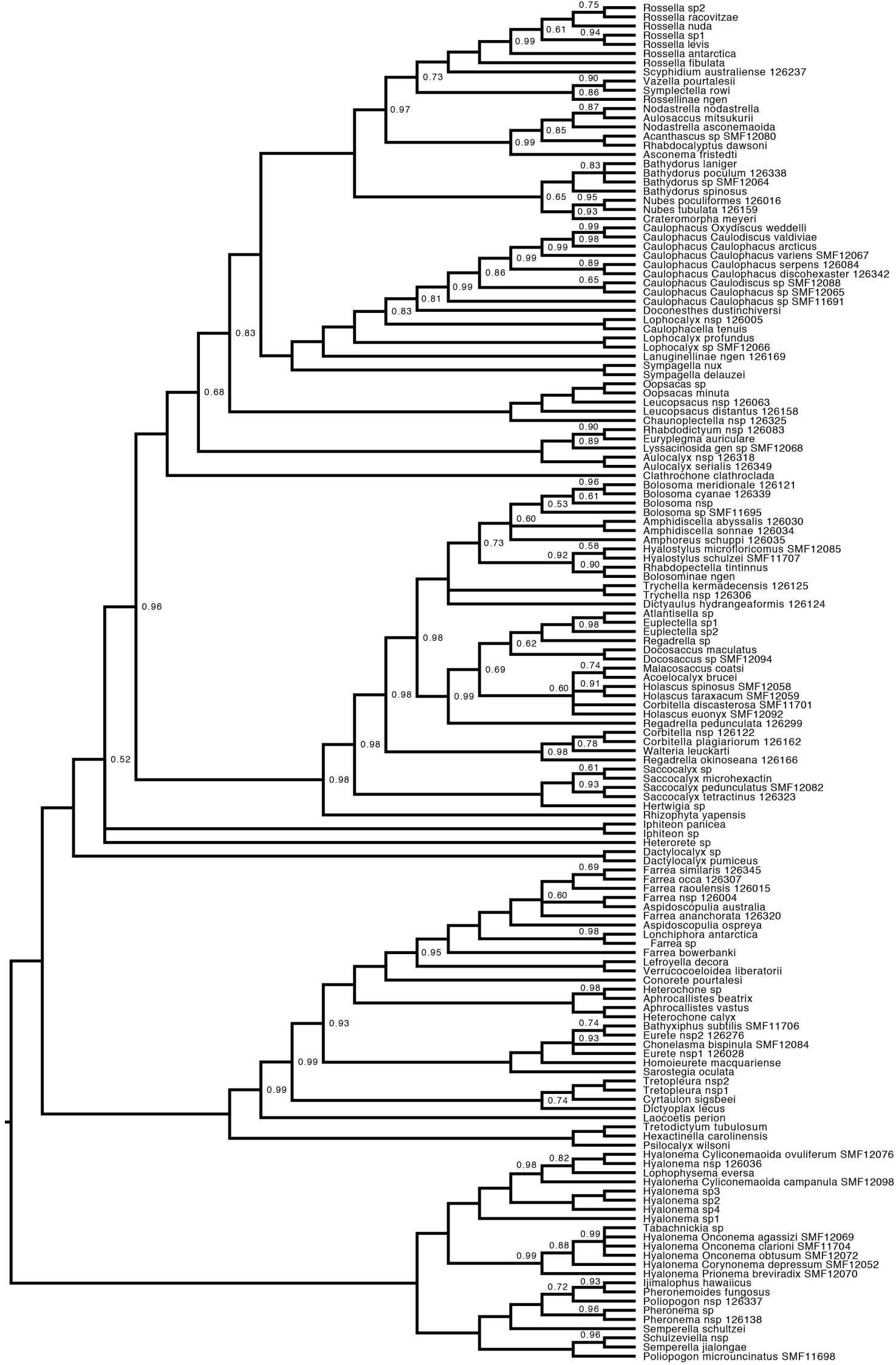

Supplement: Supplemental Information 6 — 50% majority-rule consensus cladogram. PP values ¡ 1.00 shown at nodes. [file peerj-11-15017-s006.pdf]

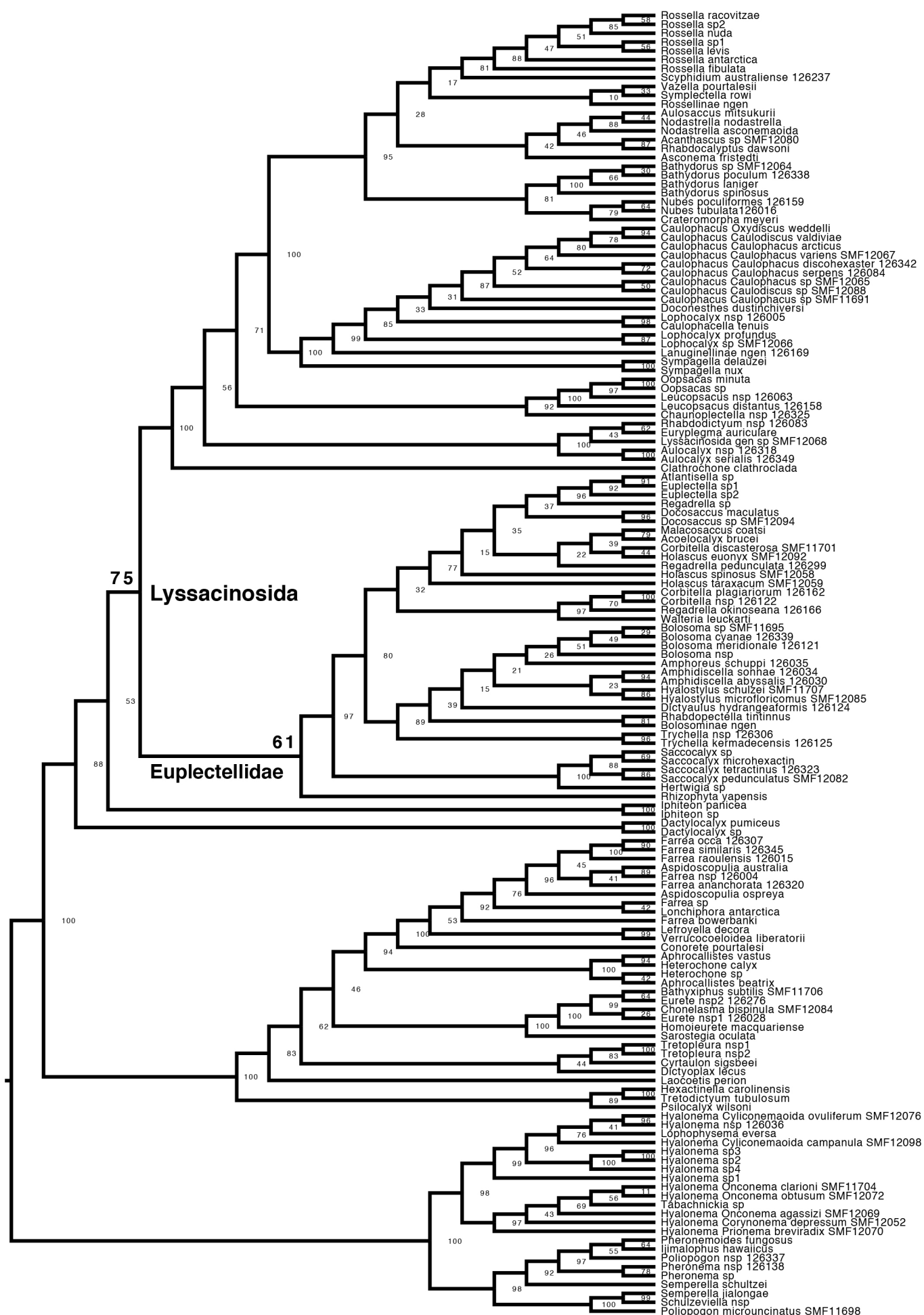

Supplement: Supplemental Information 7 — BS values for crown Lyssacinosida and crown Euplectellidae highlighted. [file peerj-11-15017-s007.pdf]

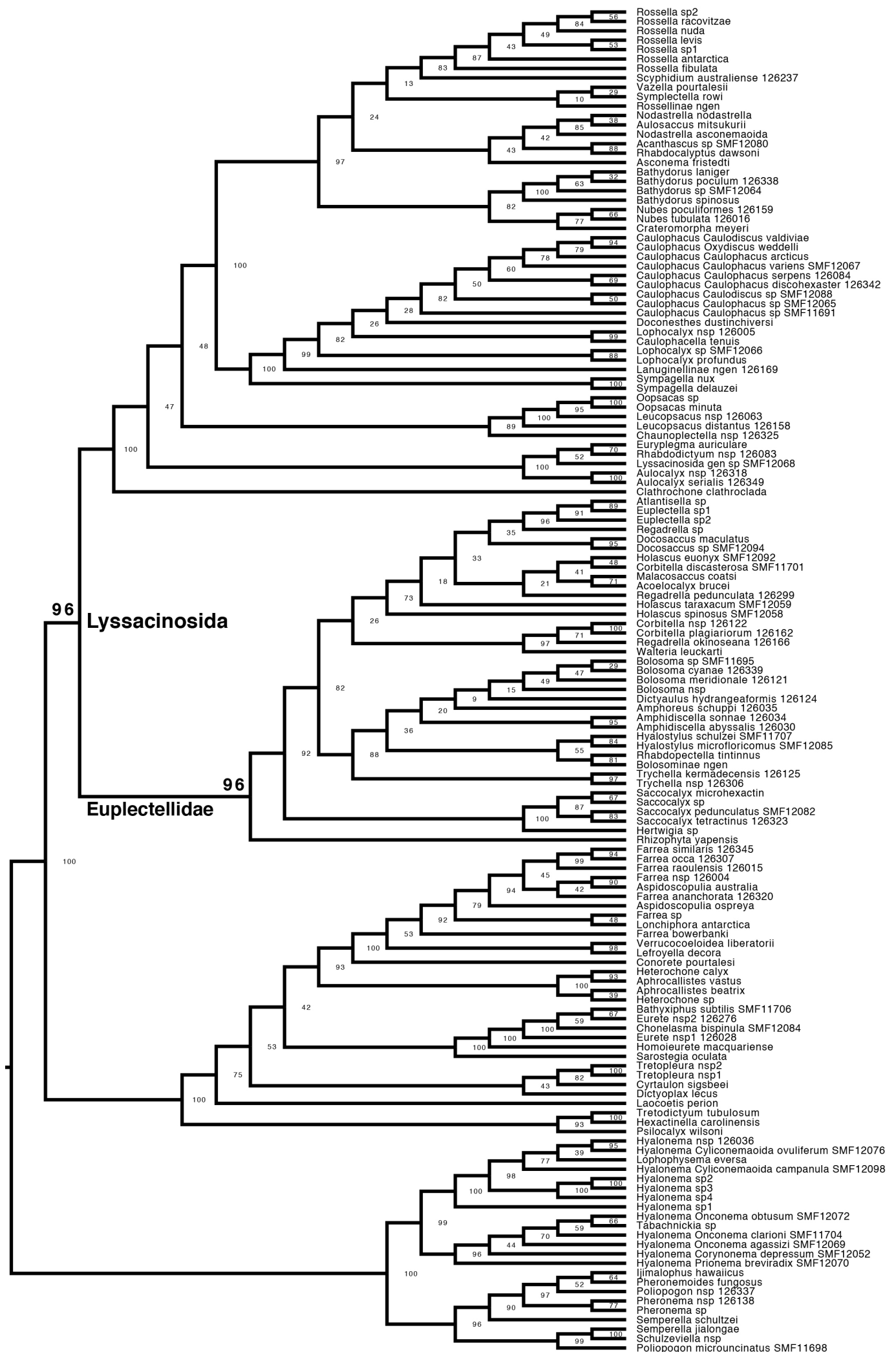

Supplement: Supplemental Information 8 — BS values for crown Lyssacinosida and crown Euplectellidae highlighted. [file peerj-11-15017-s008.pdf]
